# The association between working memory precision and the nonlinear dynamics of frontal and parieto-occipital EEG activity

**DOI:** 10.1038/s41598-023-41358-0

**Published:** 2023-08-31

**Authors:** Wen-Sheng Chang, Wei-Kuang Liang, Dong-Han Li, Neil G. Muggleton, Prasad Balachandran, Norden E. Huang, Chi-Hung Juan

**Affiliations:** 1 Institute of Cognitive Neuroscience, College of Health Sciences and Technology, National Central University, Taoyuan City, Taiwan; 2 Cognitive Intelligence and Precision Healthcare Center, National Central University, Taoyuan City, Taiwan; 3 Institute of Cognitive Neuroscience, University College London, London, UK; 4 Department of Psychology, Goldsmiths, University of London, London, UK; 5 Data Analysis and Application Laboratory, The First Institute of Oceanography, Qingdao, China; 6 Department of Psychology, Kaohsiung Medical University, Kaohsiung, Taiwan; 7 Taiwan International Graduate Program in Interdisciplinary Neuroscience, National Cheng Kung University and Academia Sinica, Taipei, Taiwan

**Keywords:** Cognitive neuroscience, Intelligence

## Abstract

Electrophysiological working memory (WM) research shows brain areas communicate via macroscopic oscillations across frequency bands, generating nonlinear amplitude modulation (AM) in the signal. Traditionally, AM is expressed as the coupling strength between the signal and a prespecified modulator at a lower frequency. Therefore, the idea of AM and coupling cannot be studied separately. In this study, 33 participants completed a color recall task while their brain activity was recorded through EEG. The AM of the EEG data was extracted using the Holo-Hilbert spectral analysis (HHSA), an adaptive method based on the Hilbert-Huang transforms. The results showed that WM load modulated parieto-occipital alpha/beta power suppression. Furthermore, individuals with higher frontal theta power and lower parieto-occipital alpha/beta power exhibited superior WM precision. In addition, the AM of parieto-occipital alpha/beta power predicted WM precision after presenting a target-defining probe array. The phase-amplitude coupling (PAC) between the frontal theta phase and parieto-occipital alpha/beta AM increased with WM load while processing incoming stimuli, but the PAC itself did not predict the subsequent recall performance. These results suggest frontal and parieto-occipital regions communicate through theta-alpha/beta PAC. However, the overall recall precision depends on the alpha/beta AM following the onset of the retro cue.

## Introduction

WM enables us to store and utilize information without external stimuli for a brief period. Previous studies have associated WM performance with oscillatory activities and interareal connectivity between frontal and posterior brain regions^1–4^. Specifically, frontal theta (4–8 Hz) and posterior alpha/beta (8–30 Hz) activities have been widely studied as indicators of WM performance^5–10^. High frontal theta power is associated with increased executive control demands in memory tasks, with larger power indicating higher cognitive requirements^11–13^. The directionality of parieto-occipital alpha/beta power, however, has shown inconsistent findings in relation to WM performance^10^. In general, suppression of alpha/beta power is associated with processes involved in forming and maintaining active WM representations, while an increase in alpha/beta power reflects the protection of memorized information from irrelevant stimuli^8,14–18^. The direction of parieto-occipital alpha/beta power modulation depends on various factors, including the specific WM task employed, the definition of the target, and the memory stages being investigated^10^. Recent advances in electrophysiological WM research have focused on interactions across different frequency components and brain areas^19–24^. Measures of cross-frequency coupling (CFC) are often used to quantify these WM-related cross-frequency interactions, e.g., the phase-amplitude coupling (PAC) between fast and slow oscillations^25,26^. In PAC, fast and slow oscillatory components are filtered from the raw signal, then the phase of the slower component is compared to the amplitude of the faster component with synchronous measures. Typically, stronger PAC is associated with greater WM demands or better WM performance^19,23,27–29^.

Although frontal theta and parieto-occipital alpha power modulations during WM maintenance have been frequently reported, their coupling is rarely discussed. Indirect evidence (e.g., Granger causality) has shown that frontal to parieto-occipital theta connectivity is transiently modulated by incoming executive demands, whereas parieto-occipital to frontal alpha/beta connectivity is sustained throughout the task procedure^20,22^. One recent dual-task study reported that frontal-midline theta power predicted the subsequent parieto-occipital alpha power lateralization. In addition, enhanced coupling was observed between frontal theta phase and parieto-occipital alpha amplitude in the cued region^24^. Nevertheless, the correlation between frontoparietal theta-alpha PAC and subsequent recall performance remained unexplored.

The standard PAC methods are increasingly criticized due to potential confounding factors arising from the nonstationary and nonlinear nature of biological signals^30–32^. The most fundamental issue comes from the physical meaning of PAC, which is a multiplicative process that cannot directly be measured with additive time–frequency decomposition methods such as short-time Fourier and wavelet decompositions^33,34^. The same limitation holds for bispectral measures frequently used to study the interactions between various frequency components of a signal^35,36^. In these methods, the amplitude modulation (AM) of a fast wave is measured by its projection to a slow reference wave. Therefore PAC and AM are inseparable, and the rhythmic AM of a fast wave cannot be measured if the frequency-matched slow wave does not present in the observed data. Such constraint limits the scope of studying the nonlinear properties of oscillatory signals.

The study's primary purpose is to investigate how nonlinear interactions of EEG oscillations are associated with WM performance. Following previous literature, we hypothesized that frontal theta and parieto-occipital alpha oscillations communicate through PAC during WM. The communication process leads to parieto-occipital alpha AM, and the pattern of AM predicts performance. Participants took part in a color recall task with simultaneous EEG recording. On each trial, they were required to remember a set of color squares. During WM maintenance, a retro cue was presented to indicate the target item. Participants responded by matching the target color with a color wheel presented one second after the cue onset. Memory precisions under different WM loads served as an index of WM performance^37,38^. The observed EEG data was decomposed with *Holo-Hilbert spectral analysis* (HHSA), which can directly measure the energy of AM in nonlinear signals^33^. In HHSA, the AMs of signal carriers are decomposed into different amplitude-modulating frequencies (AMF). The time profile of signal carriers and their AM structure are expressed in Holo-Hilbert spectra (HHS). To verify the hypothesis, we first replicated previous findings on the effect of load manipulations in EEG signals and their correlation to individual differences in WM performance. After confirmation, we investigated whether AM could reveal more information. The load effect for frontoparietal theta-alpha PAC and its correlation to WM precision were also examined for an explicit communication mechanism between frontal and parieto-occipital regions.

## Methods

### Participants

Thirty-three university students were recruited to participate in the experiment (21 males and 12 females, mean age = 23.13 years and standard deviation = 5.16 years). The sample size was determined following conventional EEG studies^7^. The recruiting process stopped when the sample size reached 30. Three more participants registered for the study before the termination of the recruitment, thus their data were also collected. All participants had a normal or corrected-to-normal vision. All participants gave informed consent before participation. The study procedures were approved by the local IRB committee (National Taiwan University Research Ethics Committee, 201606ES031). All methods were carried out in accrodance with the relevant guideliines and regulations.

### Experimental design

The behavioral task was modified from the color recall tasks for WM precision^37,38^. The stimuli were presented on an LCD monitor on a black background at a viewing distance of 60 cm. The colors were selected from a circle in CIELAB space^39^, centered at L = 70, a = 20, b = 38, in CIE coordinates with a radius of 60. The items to be remembered were a set of color squares with a size 2° × 2° of visual angle. The color of each item was sampled from 45 possible color values at every 8° of the circle. The locations of the items were randomly chosen but equally spaced concentrically, 4.5° away from the center of the monitor. The color wheel for color recall was constructed with 360 color values, sampled from 1° to 360° of the circle in the color space. The color wheel was centered on the screen, with an inner and outer radius of 6° and 8.2° of visual angle.

**Figure 1:**
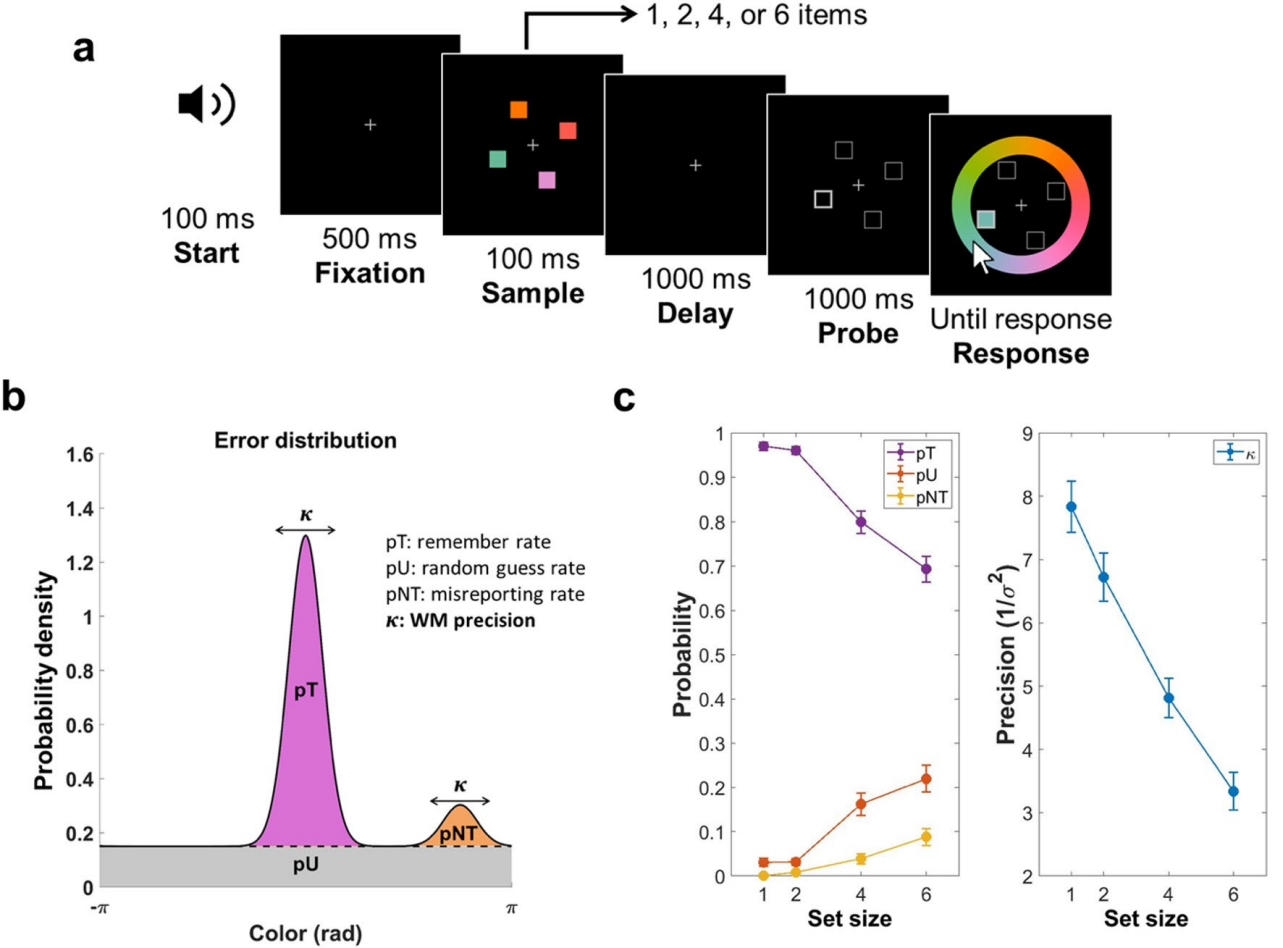
Design and behavioral results of the color recall task. (**a**) Participants were instructed to remember the color of each square in the sample array. After a 1000-ms delay period, a set of framed squares were presented to indicate sample locations. The target item was defined as the one with bold edges, and participants had to match the target color with a color wheel. During the response period, the color at the target location kept updating according to the selected color from the mouse cursor. The set size of the sample array was manipulated randomly in four conditions (1, 2, 4, or 6 items, interleaved design). (**b**) A mixture model of three components modeled the response error distribution of each condition. First, the memory of item colors was stored with noise; therefore, the response error distribution could be explained by a von Mises distribution with concentration number $\upkappa$ (reciprocal of the error variance, $1/{\sigma }^{2}$), centering at the target color location (*purple*). Since the target was not determined during the sample array, all item colors were assumed to be encoded with equal precision. Second, there was a probability of misreporting non-target colors. In this case, the variability of the response errors was explained by a set of von Mises distributions centering at the location of non-target colors with the same κ value as the remembered situation (*orange*). Third, when participants made random responses, the errors would be uniformly distributed (*gray*). Ratios of the three components could be estimated by two scalars pU (the probability of random guessing) and pNT (the probability of misreporting non-target colors). The probability of correctly remembering the target color, pT, equaled 1-pU-pNT. The results of the estimated mixture-model parameters were illustrated in (**c**) and (**d**). (**c**) pT decreased (purple line; *F*_*(3,96*)_ = 55.52, *p* < 10^–5^; *η*^2^ = 0.6344), whereas pU (red line; *F*_*(3,96)*_ = 26.20, *p* < 10^–5^; *η*^2^= 0.4501) and pNT (green line; *F*_*(3,96)*_ = 12.05, *p* < 10^–5^; *η*^2^ = 0.2764) increased as set size increased. (**d**) WM precision was defined as the concentration number $\upkappa$. $\upkappa$ decreased (*F*_*(3,96)*_ = 48.54, *p* < 10^–5^; *η*^2^ = 0.6027) as set size increased.

Each trial began with a concurrent presentation of a beep sound (500 Hz) and a central fixation cross (gray, 1°$\times$ 1°). The beep sound lasted for 100 ms, and the fixation cross remained on display throughout the trial. After 500 ms, a set of color squares was displayed for 100 ms, followed by a 1000 ms delay period. After the delay period, a set of outlined squares was presented at the location of each item during stimulus presentation for 1 s (the probe period). One of the squares had thicker edges, indicating the target item for the observer to recall. A color wheel was displayed as the probe squares vanished. The observer had to identify the target color by mouse-clicking the corresponding color on the color wheel. During the response period, the color in the target location kept updating to the color selected by the mouse cursor (Fig. 1a). The subsequent trial began 500 ms after a target color was picked from the color wheel. In different experimental conditions, the set size of the sample array varied between 1, 2, 4, or 6 items. The four set-size conditions were randomly interleaved across trials. All participants performed 90 trials for each condition, making 360 trials in total.

### Mixture model analysis

Behavioral performance was assessed using the mixture model analysis^37^. In each trial, the degree of error was obtained by calculating the angular deviation between the coordinates of the reported color and the correct target color in the color wheel. The model proposed three sources of WM errors: (1) the probability that the target color was not stored in the WM system, (2) the probability of misreporting a non-target color instead of the target color, and (3) information was stored with noise in WM. Participants were assumed to make a uniformly random response when they could not recall the target color. In addition, all items were assumed to be encoded with equal resolution and encoding to decay at an equal rate. The probability of the reported color value in each trial can therefore be described as follows:

$$p\left(x\right)=\left(1-pU-pNT\right){\Phi }_{\sigma }\left(x-\theta \right)+pU\frac{1}{2\pi }+pNT\frac{1}{m}\sum_{i=1}^{m}{\Phi }_{\sigma }\left(x-{\theta }_{i}^{*}\right)$$

where ${\Phi }_{\sigma }$ represented the von Mises (circular Gaussian) distribution with mean zero and standard deviation $\upsigma$, $x$ was the reported color value, $\theta$ was the target color value, ${\theta }_{i}^{*}, i\in \{1,\dots ,m\}$ were the *m* non-target color values, $pT$ was the probability of correctly remembering the target color, $pU$ was the probability of random guessing, and $pNT$ was the probability of misremembering the target location (Fig. 1b). The index for WM precision ($\kappa =1/{\upsigma }^{2}$) was the concentration number of the von Mises distribution, which was the reciprocal of error variance.

### EEG recording

EEG was continuously recorded throughout the experiment using SynAmps2 amplifiers (Compumedics Neuroscan) using Ag/AgCl electrodes mounted in a 64-channel electrode cap (Quik-Cap, Neuromedical Supplies). The EEG cap followed the international 10–20 system for electrode placement. Impedances of all electrodes were kept below 5 kΩ. The sampling rate was set at 2000 Hz. The horizontal and vertical electrooculograms were recorded along with the EEG recordings. AFz served as the ground electrode, whereas Cz served as the online reference. The recordings were filtered with a low-pass filter of 500 Hz.

### EEG data preprocessing

**Figure 2:**
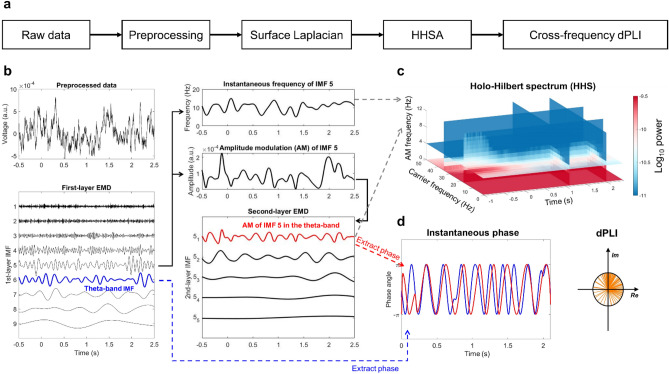
The overview of EEG data analysis. (**a**) The pipeline of EEG data analysis. EEG recordings were preprocessed with regular procedures as described in the main text. The preprocessed data were mapped into its scalp current density using the surface Laplacian, then subjected to HHSA. The phase of a slow oscillation and the phase of frequency-matching AM power of a faster oscillation could be directly obtained with HHSA. Therefore, the PAC between two oscillations could be measured with synchronization indices such as dPLI. (**b**) The procedure of HHSA involved two layers of EMD. The input signal was decomposed with the first-layer EMD to extract the oscillations of interest (e.g., IMF 5 captured the alpha-band activity). The instantaneous frequency and envelope (i.e., AM) of IMFs could be obtained with direct quadrature. The envelope of the interested oscillation was further decomposed with the second-layer EMD, yielding another set of IMFs. The second-layer IMFs represented different modes of AM oscillations of the envelope. (**c**) The Holo-Hilbert spectrum (HHS) is a joint representation of signal energy in respect of time, carrier frequency, and AM frequency. (**d**) PAC was measured with a phase synchronization index (e.g., dPLI) between the phase-providing slower oscillation (blue) and the frequency-matched AM component from the faster oscillation (red).

The raw EEG data were offline-referenced to the average of M1 and M2 electrodes. The data were epoched from – 1500 to 3000 ms relative to the onset of the sample array. Epochs containing artifacts of muscle activity, head or body movements, and electrode noise were rejected by visual inspection. Eye movements and blink artifacts were identified and removed with independent component analysis using the EEGLAB toolbox ^40^. Epochs deviating more than 200 μV were also rejected. Electrodes containing more than 50% of such ‘bad’ trials were replaced by spline interpolation of their neighbors. 19 out of 33 participants required such interpolation (three channels for one participant, two channels for four participants, and one channel for 14 participants). Most removed electrodes were located in the forehead or temporal sites (FP1/2, FPz, T7/8, TP7/8, and C6). After this procedure, none of the participants was rejected for further analysis. The averaged survived epoch ratio for all participants is 82.9% (298 trials) with 9.4% standard deviation. The preprocessed EEG signal was further transformed into its scalp current sources via the surface Laplacian method^41^. After applying the surface Laplacian transformation, the resulting signals underwent HHSA for time–frequency decomposition and phase-based statistics (Fig. 2a).

### Holo-Hilbert Spectral Analysis (HHSA)

The preprocessed EEG data were decomposed with HHSA using in-house MATLAB codes. The codes for HHSA are available in the public repository (http://osf.io/243ps). The HHSA involves two layers of nested EMD and a 3D spectral representation for the signal^33^. EMD is an iterative process that decomposes data into a set of intrinsic mode functions (IMF, Huang et al. 1998). Each IMF is symmetric locally with zero mean by definition. Therefore, the instantaneous phase can be obtained directly with the Hilbert transform or later proposed methods such as normalized Hilbert transform or direct quadrature (DQ, Huang et al. 2009). Compared to conventional approaches, HHSA has the following advantages: (1) it is not limited to the uncertainty principle of time–frequency analysis (i.e., the trade-off between time and frequency resolutions) by providing instantaneous frequency^42,43^, so the data can be represented in high precision in the spectral domain; (2) the structure of data itself determines the timescales being examined, so a priori assumptions for frequency bands are not needed; (3) the envelope function of each frequency component can be decomposed again into a set of second-layer IMFs, where each second-layer IMF represents modulation of the envelope function within a dyadic timescale; (4) EMD is an additive decomposition method; therefore the original signal can be reconstructed by direct summation over IMFs.

In HHSA, the first-layer IMFs are obtained by applying EMD to the input data (Fig. 2b, the left column). In practice, EMD acts as a dyadic filter bank to the data so that each IMF represents oscillatory activity in distinct log-2 timescales^44,45^. For instance, in an EEG signal with a 500-Hz sampling rate, the frequency range for the first IMF would be a Gaussian-like bandpass filter centering at around 128 Hz; the second IMF peaks at 64 Hz, and so on for consecutive IMFs (Fig. 4a). Since the envelopes of the first-layer IMFs are also broadband signals, they can be decomposed again with EMD (Fig. 2b, the right column). The second-layer IMFs describe the variation of envelope functions in distinct log-2 timescales; hence we obtain a representation for each envelope in the frequency domain. To distinguish the instantaneous frequencies of the first- and second-layer IMFs, we will refer to instantaneous frequencies of the first-layer IMFs as “carrier frequency” (CF) and to second-layer IMFs as “AM frequency” (AMF). The *Holo-Hilbert spectrum* (HHS) is the 3D representation of a signal over time (*t*), CF, and AMF (Fig. 2c). Extending the time–frequency representation with AMF shows the energy fluctuation induced by multiplicative interactions. Note that the AMF domain describes the results of multiplicative interaction from all possible sources. The PAC between any two signals can be evaluated via phase synchronization indices between the first- and second-layer IMFs (Fig. 2d). For instance, the theta-alpha PAC can be evaluated by calculating the directed phase-lag index (dPLI)^46^ between the theta-band carrier and the frequency-matched AM component of the alpha-band envelope. The difference between AM and PAC under HHSA is illustrated in the supplementary files (Fig. S1).

**Figure 3:**
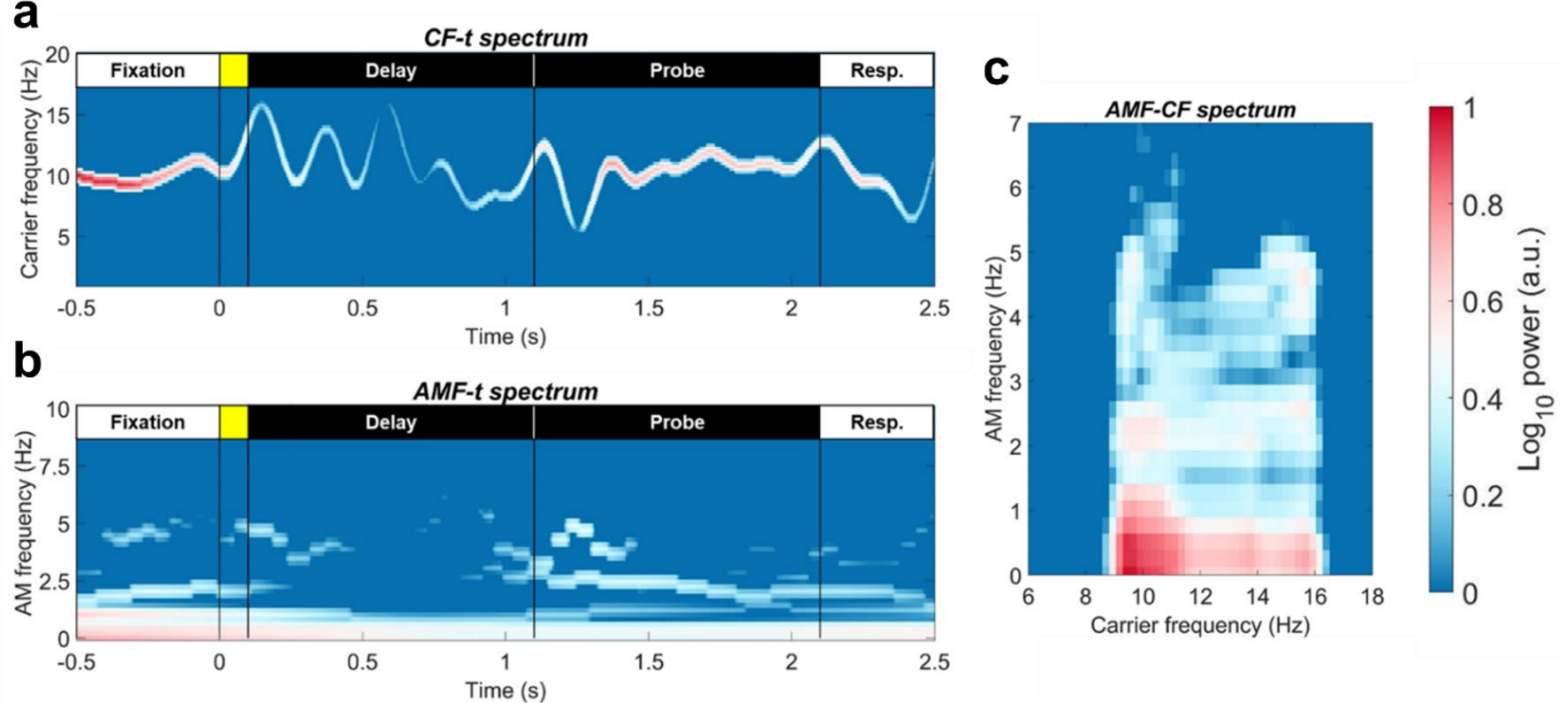
An illustrative example of spectral representations using HHSA. The example is generated with the alpha-band IMF, as illustrated in Fig. 2. Each spectrum highlights different characteristics of the signal. (**a**) The *CF-t* spectrum shows the power and frequency variations of the alpha-band IMF over time. It is similar to spectrograms obtained using classical methods (e.g., wavelet transforms) but with higher time–frequency resolutions. (**b**) The *AMF-t* spectrum shows the AM power at various modulating frequencies over time. (**c**) The *AMF-CF* spectrum shows the power distribution of carrier frequencies and their AM (in different modulating frequencies) within a given period.

In this study, HHS is projected along the three dimensions to highlight different aspects of the full spectrum. Projecting the HHS onto the *CF-time* plane created the *CF-t* spectrum (Fig. 3a), which captured the intra-mode frequency variation of the carriers. *CF-t* spectrum was equivalent to the spectrogram obtained from the Hilbert-Huang Transform (HHT)^42^ that has been frequently used for analyzing the instantaneous frequency and amplitude variations of biological signals (e.g.,^47,48^). When HHS was projected onto the *AMF-time* plane, the result highlighted the dynamic of cross-scale interaction for a given range of carrier frequencies (the *AMF-t* spectrum, Fig. 3b). When HHS was projected onto the *AMF-CF* plane, the results showed the overall structure of cross-scale interactions between different frequency components in a given period (the *AMF-CF* spectrum, Fig. 3c). In this study, the resolution of HHS was set as 4 ms for time and 0.5 Hz for both CF and AMF.

Transient bursts in biological signals sometimes cause mode mixing, i.e., a single IMF contains multiple frequency components with classical EMD. Here, we applied an improved version of EMD utilizing sinusoidal masking signals in conjunction with the original EMD algorithm to reduce the problem^47,49,50^. A set of masking signals with initial phases 0, π/2, π, and 3π/2 were added into a pre-decomposed IMF obtained from regular EMD to enhance decomposition. The optimal amplitude of the masking signals is 1.6 times above the average amplitude of the components through experience^50^. We used a factor of 2 for both layers of EMD. The frequency of masking signals was the same as the mean instantaneous frequency of the pre-decomposed IMF. Instantaneous frequencies of IMFs were directly calculated by taking time derivatives of instantaneous phases obtained from DQ.

### Intra- and inter-areal PAC under the HHSA framework

IMF is a function that requires (1) symmetric locally to zero, and (2) there exists only one extremum between two successive zero-crossing points. According to the above definition, the instantaneous phases for the first- and second-layer IMFs can be obtained directly with DQ. Given the phase functions of carriers and their AMs, the degree of PAC can be measured by phase synchronization indices. Here, we used the dPLI to quantify the degree of PAC while taking the leading-lagging relationship into account:

$${\mathrm{dPLI}}_{fg}= \frac{1}{N}\sum_{n=1}^{N}{H(Im(e}^{i\left({\varphi }_{f}\left(n\right)-{\varphi }_{g}\left(n\right)\right)})),$$

where ${\varphi }_{f}$ and ${\varphi }_{g}$ denote the phase functions of two oscillations, and *N* denotes the number of sampling points within a time window. *H* is the Heaviside function (H(x) = 0 if x ≤ 0; H(x) = 1 if x > 0), and $Im$ denotes that only the imaginary part of the relative phase is considered. The dPLI is a directed synchronization measure bounded by 0 and 1. If signal *f* is phase-leading to signal *g*, then ${0.5<\mathrm{dPLI}}_{fg}\le 1$; if signal *g* is phase-leading to signal *f*, then ${0\le \mathrm{dPLI}}_{fg}<0.5$. When PAC employs the dPLI formula under the HHSA framework, one phase function is from the carrier of a slower oscillation, and the other is from the frequency-matched AM component of a faster oscillation. This approach is similar to the Holo-Hilbert cross-frequency phase clustering (HHCFPC) that has been demonstrated as efficient in quantifying intra- and inter-areal CFC in the process of WM^21^.

Combining HHSA and phase-based statistics to evaluate PAC has the following advantages over conventional methods: first, phase-based statistics, such as the phase-lag index, only consider the imaginary part of the spectrum. Therefore, they are robust to noise. Second, nonstationarity in the high-frequency power leads to instability in the Hilbert spectrum and is resolved via EMD. Third, different modulating waves may interfere when the modulation strength is determined by the distribution of high-frequency amplitude over low-frequency phase bins (e.g.,^26^). In summary, the confounding of power is eliminated by utilizing phase-based statistics. The waveform shape is essentially the nonstationarity issue, which is resolved by EMD. The phase-reset factor is related to separating co-occurring phenomena from actual modulation.

### Statistical analyses of EEG data

We used MATLAB and the FieldTrip Analysis Toolbox for the statistical analysis of EEG data^51^. Epochs were time-locked to the onset of the sample array for all analyses.

#### Evaluating the effect of WM load on EEG activity

For preliminary analysis, we calculated the mean amplitude of each IMF across all electrodes for each set size and participant, then tested the effect of load manipulation with a repeated-measures ANOVA for set sizes 2, 4, and 6 at each time point. The single-item condition served as active control for the task. ANOVA results were corrected for multiple comparisons by a nonparametric cluster-based permutation test of 5000 iterations. The method only assumes that every experimental condition is associated with a probability distribution. Unlike parametric tests, the validity of the permutation test does not depend on the probability distribution of the data or the cluster-forming method^52^. Both the cluster-forming threshold and the critical value of significance were set at *p* = 0.05.

Based on previous studies^6,7,24^, we choose mid-frontal electrodes (AF3/4, F1/2/3/4, FC1/2/3/4, Fz, and FCz) and parieto-occipital electrodes (P1/2/3/4/5/6/7/8, PO3/4/5/6/7/8, O1/2, Pz, POz, and Oz) for area-based analysis. Averaged *CF-t* power spectra in frontal and parieto-occipital electrodes were calculated separately, ranging from 1 to 50 Hz. Load effects for the *CF-t* spectra were evaluated with the same procedure of repeated-measures ANOVA as the preliminary analysis. If any significant cluster was identified, the *AMF-t* spectrum of the corresponding CF range was calculated and tested with the same ANOVA procedure.

#### Correlation between individual WM precision and EEG activity

The same *CF-t* and *AMF-t* representations of parieto-occipital EEG power were also used for correlation analyses. EEG spectra and $\kappa$ values from the set-size one condition were subtracted from those from set sizes 2, 4, and 6, and were then transformed into z-scores for regression analysis. For each spectrum, a general-linear model (GLM) was constructed in which the z-transformed $\kappa$ values served as a regressor to predict the z-transformed EEG power in each spectral point. Since both $\kappa$ values and EEG power varied systematically with increasing set size, the regression weights were separately evaluated for set sizes 2, 4, and 6, then averaged together for statistical tests (i.e., set size served as a control variable for the GLM). The approach was similar to previous within-subject studies^53,54^ but on a between-subject basis. The same cluster-based permutation procedure was applied to correct the results of GLM for load-effect analysis, but both cluster-forming threshold and critical value were set at *p* = 0.05 for two-tailed tests. In addition, we also tested EEG activity in frontal electrodes to see if there was any WM correlate in the cluster. Correlations between frontal *CF-t* spectrum and WM precision were analyzed first; if any significant correlate was identified, the *AMF-t* spectrum of the corresponding CF range was analyzed.

#### Intra- and inter-areal PAC

The oscillations of interest were beta, alpha, and theta waves for this study. In this dataset, the frequency ranges of IMFs 4, 5, and 6 represented oscillatory activities in the beta-, alpha-, and theta-band, respectively. The intra- and inter-regional PAC were calculated as follows: first, we obtained the phase of theta waves (from IMF 6) and the phase of frequency-matched alpha/beta AM components (from IMFs 4 and 5) through HHSA. Second, the cross-frequency dPLI between theta phase and frequency-matched alpha/beta AMs across all possible electrode pairs was calculated and then averaged together according to the frontal and parieto-occipital electrodes of interest for each set-size condition. Time windows for dPLI evaluation were determined according to the previous results of load effects over HHS. The first interval ranged from 0 to 0.5 s after the onset of the sample array, and the remaining part of the delay period constituted the second interval. Time windows for the probe period were also divided into the first 0.5 s and the remainder interval for analysis. The effect of load manipulation was evaluated separately by a repeated-measures ANOVA for different time windows and electrode clusters. In addition, the phase synchronization of frontal and parieto-occipital regions was tested by the same procedure. The overall results were corrected for multiple testing using the Benjamini–Hochberg procedure^55^ with a false-discovery rate (FDR) of 0.05.

## Results

### Behavioral results

Thirty-three participants performed a color-recall task with varying WM loads. Response errors in different loads were fitted separately by a mixture model. As expected, participants performed worse as set size increased (Fig. 1c). With increasing set size, the probability for remembered response (pT) decreased (*F*_*(3,96)*_ = 55.52, *p* < 10^–5^; Cohen’s f = 1.7352), whereas the probability for misreported responses (pNT; F_(3,96)_ = 12.05, *p* < 10^–5^; Cohen’s f = 0.3820) and guessed responses (pU; *F*_*(3,96)*_ = 26.20, *p* < 10^–5^; Cohen’s f = 0.8185) increased. On top of the decreasing recall rates, WM precision (κ) also decreased as set size increased (*F*_*(3,96)*_ = 48.54, *p* < 10^–5^, Cohen’s f = 1.5170). Post hoc analyses revealed significant differences of κ between set sizes 1 and 2 (*t*_*32*_ = − 2.728, *p* = 0.0103) but no significant difference for pT (*t*_*32*_ = − 1.2182, *p* = 0.2321), indicating that WM precision (κ) was more sensitive in small WM loads. Parameters $\upkappa$, pT, and pU show significant differences between set sizes 2 and 4 ($\upkappa$, *t*_*32*_ = − 4.7273, *p* = 4.38 × 10^–5^; pT, *t*_*32*_ = − 7.4262, *p* = 1.89 × 10^–8^; pU, *t*_*32*_ = 5.7989, *p* = 1.94 × 10^–6^), as well as 4 and 6 ($\upkappa$, *t*_*32*_ = − 3.889, *p* = 4.78 × 10^–4^; pT, *t*_*32*_ = -4.6453, *p* = 5.55 × 10^–5^; pU, *t*_*32*_ = 2.581, *p* = 0.0146) after FDR correction at 0.05 level. pNT differences between set sizes 2 and 4 (*t*_*32*_ = 2.3583, *p* = 0.0246) as well as set sizes 4 and 6 (*t*_*32*_ = 2.4185, *p* = 0.0215) were not significant after FDR correction. We performed the sensitivity analysis for the four mixture model parameters given 33 participants, 0.05 significant threshold, and 0.80 statistical power. The critical effect sizes for $\upkappa$, pT, pNT, and pU were 0.2459, 0.2345, 0.2926, and 0.2427, respectively.

### WM load modulated parieto-occipital alpha/beta power

**Figure 4:**
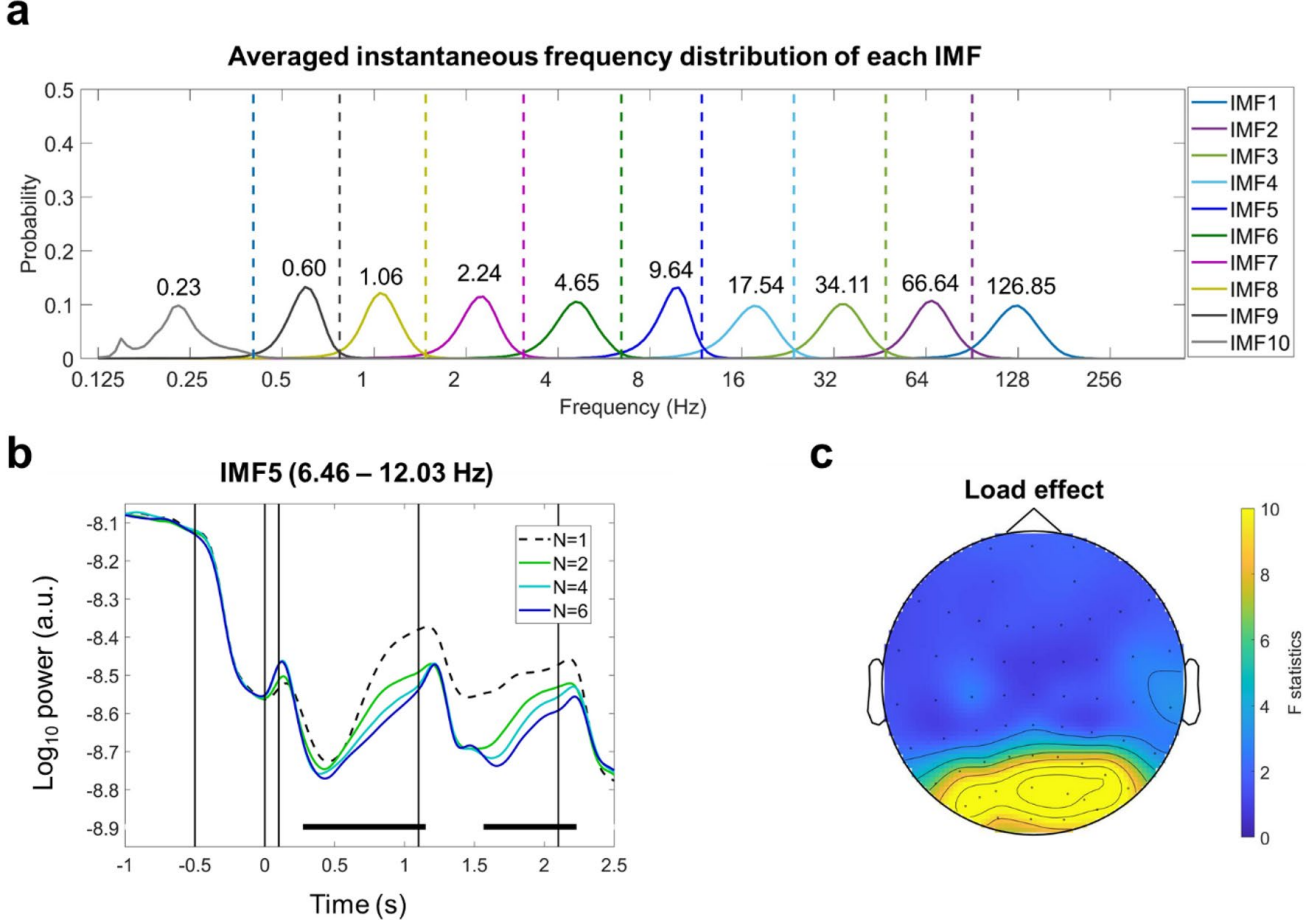
WM load modulated parieto-occipital alpha amplitude. (**a**) The instantaneous frequency distributions of IMFs averaged across all electrodes and all participants. Numbers denote the peak frequency of each IMF distribution. IMF 5 (6.46–12.03 Hz) fell within the timescale of the alpha-band frequency. (**b**) The time course of the grand average amplitude of IMF 5 across all electrodes for each set size. A repeated-measure ANOVA for set sizes 2, 4, and 6 (set-size 1 served as the baseline condition for comparison) was performed for each time point and then was corrected by a cluster-based permutation procedure of 5000 iterations. Color bars indicated cluster-corrected *p* values for each cluster. Three significant clusters were observed. A positive cluster could be identified following the sample array onset (cluster-corrected *p* = 0.057), and two negative clusters could be observed both in the delay (cluster-corrected *p* = 4 × 10^–4^) and probe periods (cluster-corrected *p* ≤ 2 × 10^–4^). (**c**) The topography of F-statistics averaged from 0 to 2.1 s after the onset of the sample array showed that the effect of load manipulation was concentrated in parieto-occipital electrodes.

The HHSA is based on the empirical mode decomposition (EMD), which decomposes the temporal signal into a set of intrinsic-mode functions (IMF, Huang et al., 1998). The IMFs serve as a dyadic filter bank for the data^50^. Each IMF represents EEG activity in a different dyadic timescale (Fig. 4a). Between-participant variations of the peak frequency and frequency range for each IMF were summarized in Table 1. IMFs 4, 5, and 6 represented the beta-, alpha-, and theta-band activities, respectively. Standard deviations of the frequency range and peak frequency were smaller than 1 Hz for the three IMFs.

**Table 1:** Descriptive statistics of peak and the lower-bound frequencies for each IMF.

IMF	Peak (Hz)		Lower bound (Hz)	
	M	SD	M	SD
1	126.85	7.04	90.02	4.29
2	66.64	3.02	47.05	1.90
3	34.11	1.42	23.68	0.95
4	17.54	0.94	12.03	0.45
5	9.64	0.79	6.46	0.35
6	4.65	0.29	3.03	0.10
7	2.24	0.08	1.46	0.06
8	1.06	0.05	0.75	0.04
9	0.60	0.04	0.41	0.02
10	0.23	0.01	N/A	N/A

The lower-bound frequencies are the maximum likelihood estimators separating two consecutive IMF distributions.

Our EEG analysis first verified whether the task used in the study replicated the load-dependent alpha power suppression in the previous literature^6,7,14,56^. For each IMF, the grand-average powers across all electrodes were calculated first, then the effect of load manipulation was evaluated by performing a repeated-measures ANOVA at each time point and each IMF. They were corrected with a cluster-based permutation procedure of 5000 iterations. The set-size 1 condition served as the baseline for comparison. The effects were quantified as the deviation from the baseline in set sizes 2, 4, and 6. Significant clusters were observed in IMFs 4 to 7, in which IMFs 4 (12.03–23.68 Hz) and 5 (6.46–12.03 Hz) showed negative load effects in the delay and probe periods, whereas IMFs 6 (3.03–6.46 Hz) and 7 (1.46–3.03 Hz) showed transient positive load effects following the onsets of sample and probe arrays (Fig. S2). For the IMF capturing alpha activity, two negative clusters could be observed within the latency of interest (-0.5 to 2.5 s relative to the onset of the sample array, Fig. 4b). The first cluster was observed in 0.5–1.15 s (cluster-corrected *p* = 4 × 10^–4^), and the second cluster was observed in 1.55–2.1 s (cluster-corrected *p* ≤ 2 × 10^–4^). A marginal positive load effect was observed following the onset of the sample array (0–0.2 s; cluster-corrected *p* = 0.057). On average, the F-statistics from 0 to 2.1 s after the onset of the sample array concentrated in parieto-occipital electrodes (Fig. 4c).

**Figure 5:**
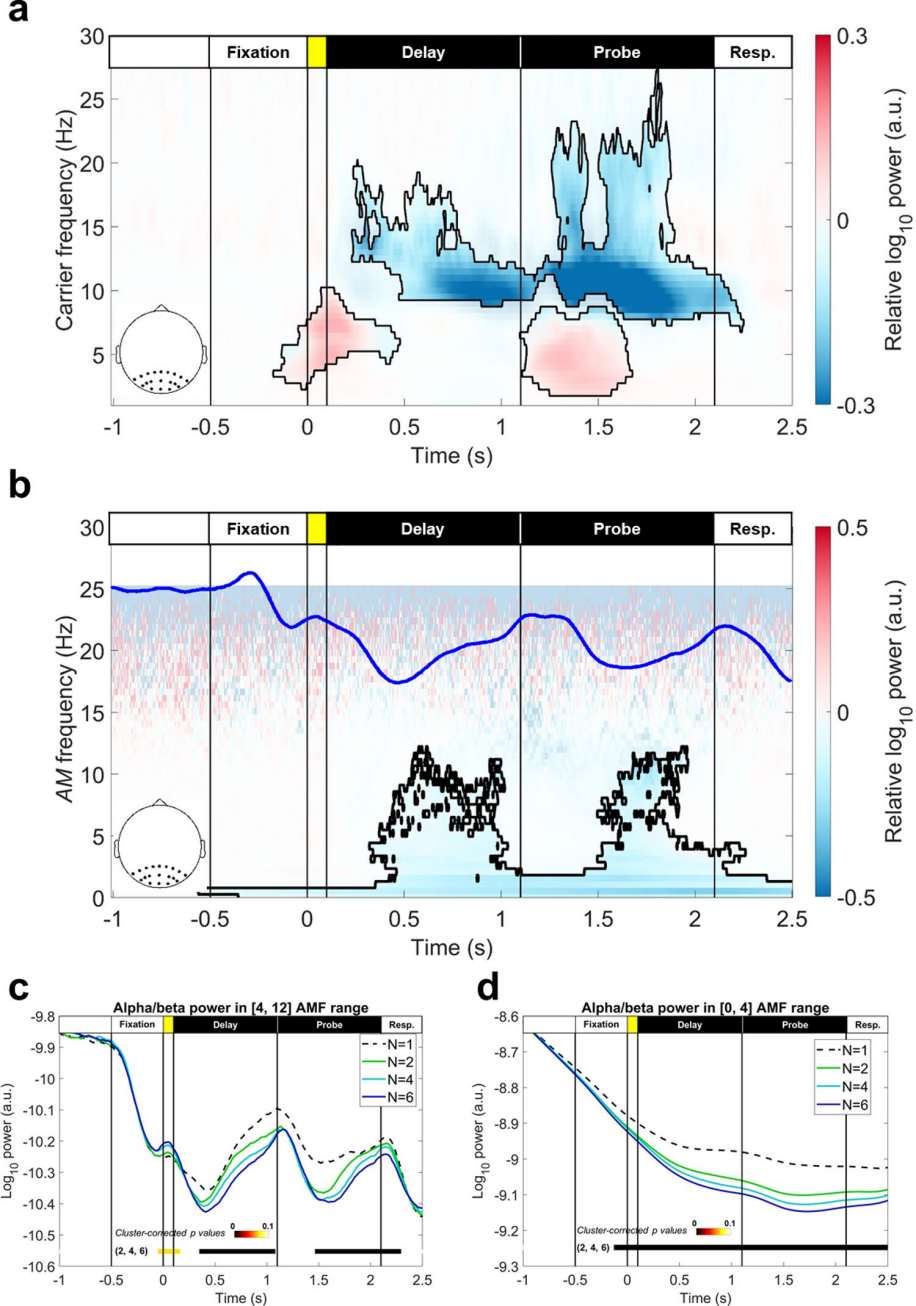
*CF-t* and *AMF-t* representations of load-dependent modulation of parieto-occipital EEG power. (**a**) Mean *CF-t* spectrum for set sizes 2, 4, and 6, contrasted with the set-size 1 condition. Load effects were verified by performing a repeated-measure ANOVA for each spectral point and were corrected by a cluster-based permutation procedure of 5000 iterations (both cluster-forming threshold and cluster significant level were set as *p* = 0.05). Black contoured areas denote significance after correction for multiple comparisons. Here, a sustained negative cluster was identified during the delay and probe periods (cluster-corrected *p* = 4 × 10^–4^), and two positive clusters could be observed after the onsets of the sample (cluster-corrected *p* = 6.2 × 10^–3^) and probe arrays (cluster-corrected *p* = 4 × 10^–4^). (**b**) The same analysis was performed for the *AMF-t* spectrum of IMFs 4 and 5 (6.4–23.6 Hz). The results showed different time courses for different AMs of sustained load-dependent power suppression. The low-frequency AMF component (0–4 Hz) extended throughout the task procedure, whereas the 4–12 Hz AMF component only showed significance after processing the sample and probe stimuli (i.e., 0.5 s after stimuli onsets, cluster-corrected *p* = 5 × 10^–4^). The blue trace showed the fluctuation (**c**) WM load modulated the fast (4–12 Hz) AMF component in 0.5 s after the onsets of the sample and probe arrays. The color bar denoted the cluster-corrected *p*-value for each cluster. (**d**) The slow (< 4 Hz) AMF component showed significant load effects throughout the maintenance period.

Next, we mapped parieto-occipital EEG activity into HHS, then verified the load effect using the same ANOVA procedure as the above. The results were similar to previous studies^7,56^. A sustained negative cluster was observed throughout the delay and probe periods in the alpha- and beta-bands (8–23 Hz; cluster-corrected *p* = 4 × 10^–4^; Fig. 5a). Compared with alpha power suppression, the effect of beta power (12–23 Hz) suppression was more pronounced in the probe period (1.1 to 2.1 s from the onset of sample array). On the other hand, transient positive clusters were observed in the theta-band (2–8 Hz) frequency during the onsets of the sample (cluster-corrected *p* = 6.2 × 10^–3^) and probe arrays (cluster-corrected *p* = 4 × 10^–4^). The observed alpha/beta power suppression was not driven by the evoked responses, since the the same result could be replicated in the induced activity (Fig. S7). The theta-band clusters, on the other hand, was more related to evoked responses.

To further quantify the time course of inter-frequency power modulation in terms of AMF for the sustained negative cluster observed, we calculated the *AMF-t* spectrum within the alpha and beta time scales (i.e., IMFs 4 and 5), then performed the same analysis as for the *CF-t* spectrum. The results showed that the sustained negative cluster observed consisted of two AMF components (cluster-corrected *p* = 5 × 10^–4^, Fig. 5b): a low-frequency component (0–4 Hz) extended throughout the task procedure (Fig. 5c), and a 4–12 Hz component that was only observed in the latter parts of the delay and probe periods (Fig. 5d). Note that the 4–12 Hz component was observed in the same time window in which the power of alpha CF gradually increased from the dips following the onsets of the sample and probe arrays (the blue trace in Fig. 5b).

We also tested the load effect for frontal EEG power, but no significant effect was observed (Fig. S3). For comparison between HHSA and traditional time–frequency analysis, a separate analysis with decomposition using the Morlet wavelet transforms was illustrated in Fig. S4. The patterns of the results were similar for the two methods. However, HHSA showed more precise time–frequency resolution, and only the HHSA could provide information in terms of AMF.

### WM precision and HHS in the parieto-occipital region

**Figure 6:**
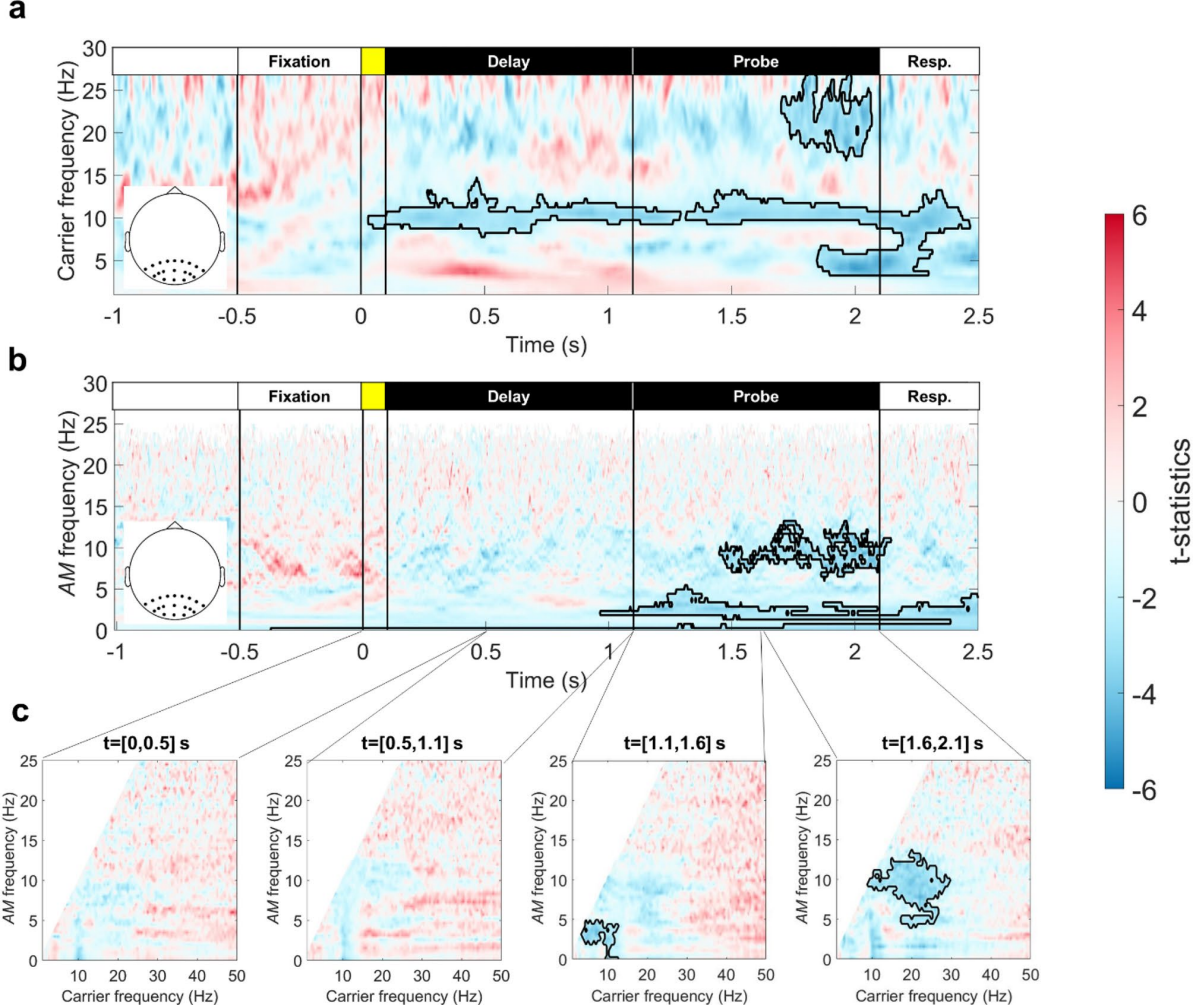
Representations of correlation strengths between parieto-occipital HHS and WM precision. Parieto-occipital alpha/beta power was negatively correlated to WM precision (κ). Black outlines denote significant correlation after cluster-based permutation correction (5000 iterations, *p* < 0.05 in a two-tailed test). (a) For the *CF-t* spectrum, a sustained negative correlation could be identified in the alpha-band (9–12 Hz) in the delay (cluster-corrected *p* = 0.007) and the probe periods cluster-corrected *p* = 0.002). In addition, a negative beta (17–26 Hz, cluster-corrected *p* = 0.013) and theta clusters (3–7 Hz, cluster-corrected *p* = 0.002) to κ value were also observed near the end of the probe period. (**b**) The *AMF-t* spectrum for the first-layer IMFs 4 and 5 (6.4–23.6 Hz) showed three AMF correlates to WM precision: an alpha-AMF correlate (7–13 Hz) was observed in the latter part of the probe period (cluster-corrected *p* = 0.0152), a delta-AMF correlate (1.5–4 Hz) was observed from the onset of probe array and was extended to the response period, and a low-frequency correlate (0–0.5 Hz) was observed throughout the task procedure (both belong to the same cluster, cluster-corrected *p* = 0.0044). (c) The results for *CF-AMF* spectra showed a negative correlation with WM precision during the probe period. In the first 500-ms time window of the probe period (t = [1.1, 1.6]), a significant cluster for theta/alpha CF was modulated in 2–5 Hz AMF (cluster-corrected *p* = 0.0106); in the last 500-ms time window (t = [1.6, 2.1]), a correlate of beta CF modulated in 5–12 Hz AMF (cluster-corrected *p* = 0.005) was identified.

After evaluating the load effects across different set sizes, we conducted a correlation analysis for the individual differences between WM precision (κ) and parieto-occipital HHS. The set-size 1 served as the baseline condition. The κ value and HHS were subtracted from the single-item condition and transformed into z-scores for analysis. The correlation was evaluated by a general linear model between the κ value and each spectral point of the HHS, with WM load as a covariate. The results were corrected with a cluster-based permutation test with 5000 iterations. The results for the *CF-t* spectrum showed a sustained negative correlation in the alpha band for both the delay (cluster-corrected *p* = 0.007) and probe periods (cluster-corrected *p* = 0.002; Fig. 6a). Beta power was negatively correlated with the κ value in the last 0.4 s time window of the probe period (cluster-corrected *p* = 0.013). In addition, theta power also negatively correlated with the κ value toward the end of the probe period (the cluster was connected to the alpha-band cluster in the response period; cluster-corrected *p* = 0.002). The same analysis with wavelet decomposition yielded similar results (Fig. S5).

For the *AMF-t* spectrum, three AMF correlates of WM precision could be identified (Fig. 6b). First, an alpha-AMF correlate (7–13 Hz) was observed in the latter part of the probe period (1.5–2.1 s from the onset of sample array; cluster-corrected *p* = 0. 0152). Second, a low-frequency AMF component (1.5–4 Hz) was observed from the onset of the probe array, and it extended to the response period. Finally, the trend component (0–0.5 Hz) showed a sustained negative correlation throughout the task procedure. The latter two correlates were connected in the response period (cluster-corrected *p* = 0.0044). Further GLM analyses for the *CF-AMF* spectra showed that the parieto-occipital alpha/beta AM predicted individual WM precision after a target-defining cue was presented to the participants (Fig. 6c).

### WM precision and HHS in the frontal region

**Figure 7:**
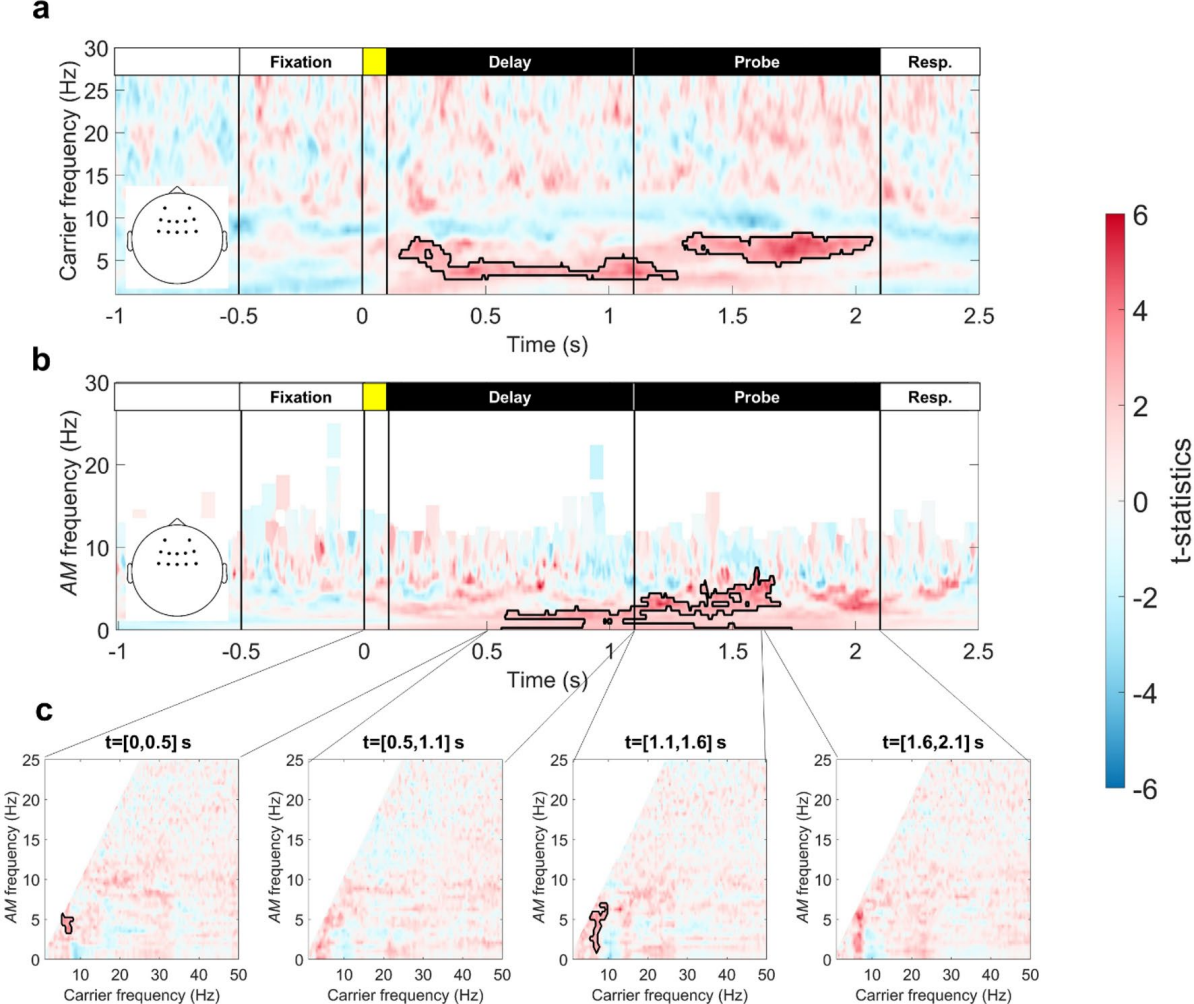
Representations of correlation strengths between frontal HHS and WM precision. Frontal theta power was positively correlated with WM precision (κ). Black outlines denote significant correlation after cluster-based permutation correction (5000 iterations, *p* < 0.05 in a two-tailed test). (**a**) The *CF-t* spectrum showed a positive correlation during WM retention, but the frequency of the correlate identified in the delay period was slower (3–5 Hz; cluster-corrected *p* = 0.0054) than the correlate identified in the probe period (5–8 Hz; cluster-corrected *p* = 0.0092). (**b**) The *AMF-t* spectrum for the first-layer IMFs 6 and 7 (1.5–11.8 Hz) showed a positive correlation with WM precision around the onset of the probe array (t = [0.5, 1.6] s). A significant positive cluster in the 1–4 Hz AMF range was observed during this time window (cluster-corrected *p* = 0.0074). (**c**) The *CF-AMF* spectra showed positive correlations with WM precision in the first 0.5-s time window after the sample array onset (modulated in 3–5 Hz AMF; cluster-corrected *p* = 0.0168) and the first 0.5-s time window after the probe array onset (modulated in 1–6 Hz AMF; cluster-corrected *p* = 0.0076).

The GLM results for frontal HHS showed a positive correlation with individual WM precision (Fig. 7). For the *CF-t* spectrum, the theta-band power was positively correlated to $\kappa$ value during WM retention. However, the frequency range of frontal correlates was slightly different in the delay and probe periods (Fig. 7a). The frequency range was 3–5 Hz for the cluster observed in the delay period (cluster-corrected *p* = 0.0054), whereas the frequency range was 5–8 Hz for the cluster observed in the probe period (cluster-corrected *p* = 0.0092). The comparable analysis with the Morlet wavelet transforms only showed a positive correlation trend, but the statistics did not pass the significant level after multiple corrections (Fig. S5).

**Figure 8:**
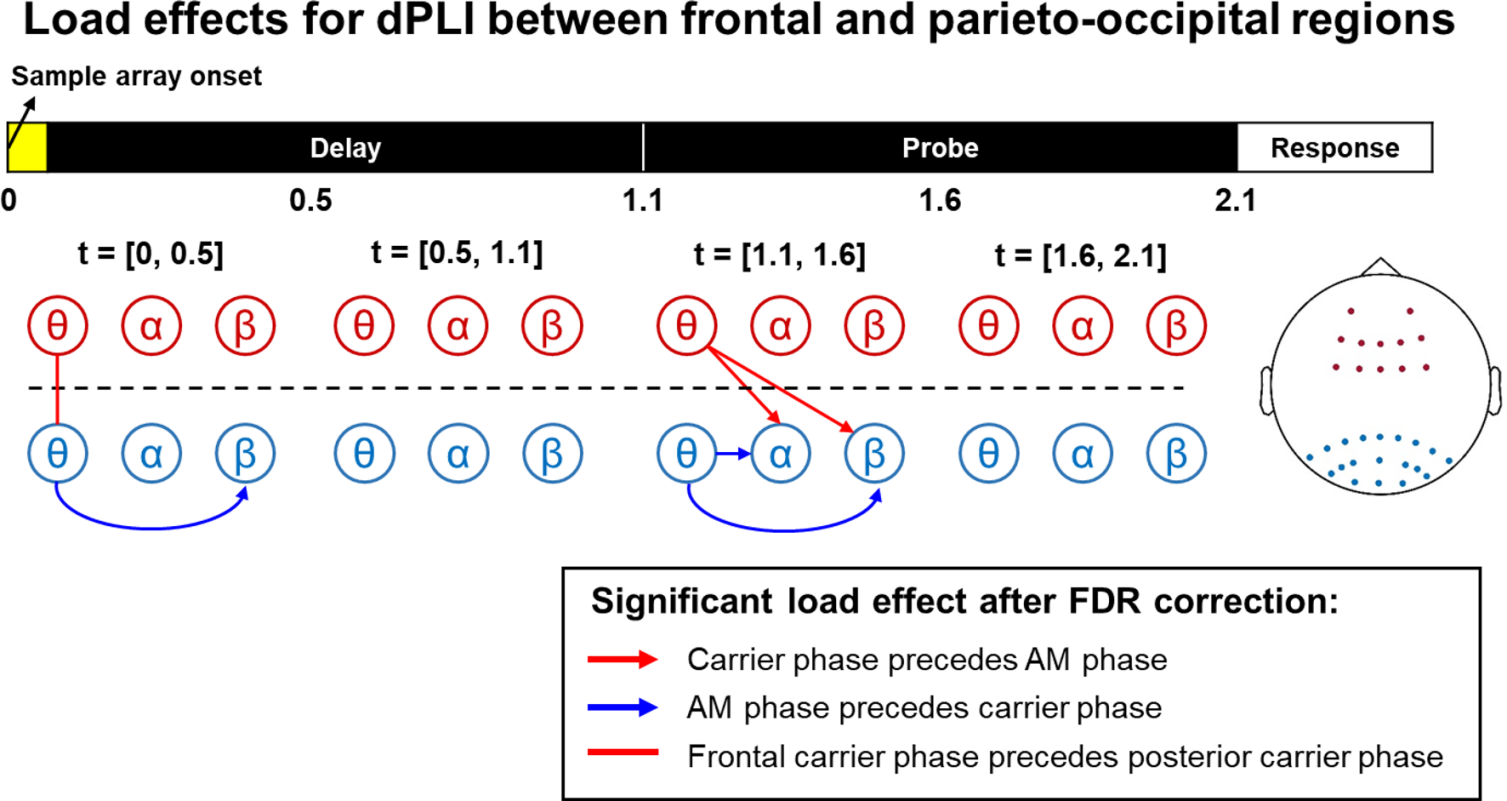
Load effects of intra- and inter-areal dPLIs between frontal and parieto-occipital electrodes of interest. Nodes represent oscillatory activity in different timescales in frontal and parieto-occipital regions. Arrows between nodes indicate significant load effects (FDR < 0.05) in cross-frequency dPLI. The arrow nock denotes the carrier phase, and the arrowhead denotes the phase of the frequency-matched AM. A vertical line denotes a significant load effect in dPLI between frontal and parieto-occipital oscillatory activities in the theta-band. Significant load effects were observed during the first 0.5-s following the onsets of the sample and probe array. All significant effects were positive. In the first interval (t = [0, 0.5] s), the long-range dPLI for theta oscillations and the scale-matched alpha/beta AM increased with the set size. In contrast, the local (within the parieto-occipital region) dPLI decreased between the theta phase and beta AM. A similar pattern re-emerged during the onset of the probe array (t = [1.1, 1.6] s). Furthermore, dPLI for frontal and parieto-occipital theta phases also increased during the onsets of the sample and probe array. All significant frontoparietal effects were positive, whereas all significant intraparietal effects were negative. Since the dPLI was 0.5 in the set-size one condition across all pairs, the pattern of results indicated that the frontal theta phase drives parieto-occipital alpha/beta AM during the encoding and retrieval of color squares.

When the frontal theta power (IMFs 6 and 7) was mapped into the *AMF-t* spectrum, a 1.5–4 Hz AMF component was observed in 0.5 s before and after the onset of the probe array (cluster-corrected *p* = 0.0074; Fig. 7b). The result reflected the transition from the lower-theta (3–5 Hz) to the upper-theta (5–8 Hz) band as observed in Fig. 5a. The results of GLM analyses for the *CF-AMF* spectra in the same area showed that the AM component of the frontal theta power showed a significant correlation during the processing of incoming stimuli. A significant cluster could be observed in the first 500-ms time window of the delay (Fig. 8c, the first diagram; cluster-corrected *p* = 0.0168) and probe periods (Fig. 7c, the third diagram; cluster-corrected *p* = 0.0076).

### Intra- and inter-regional PACs between theta and alpha/beta oscillations

**Figure 9:**
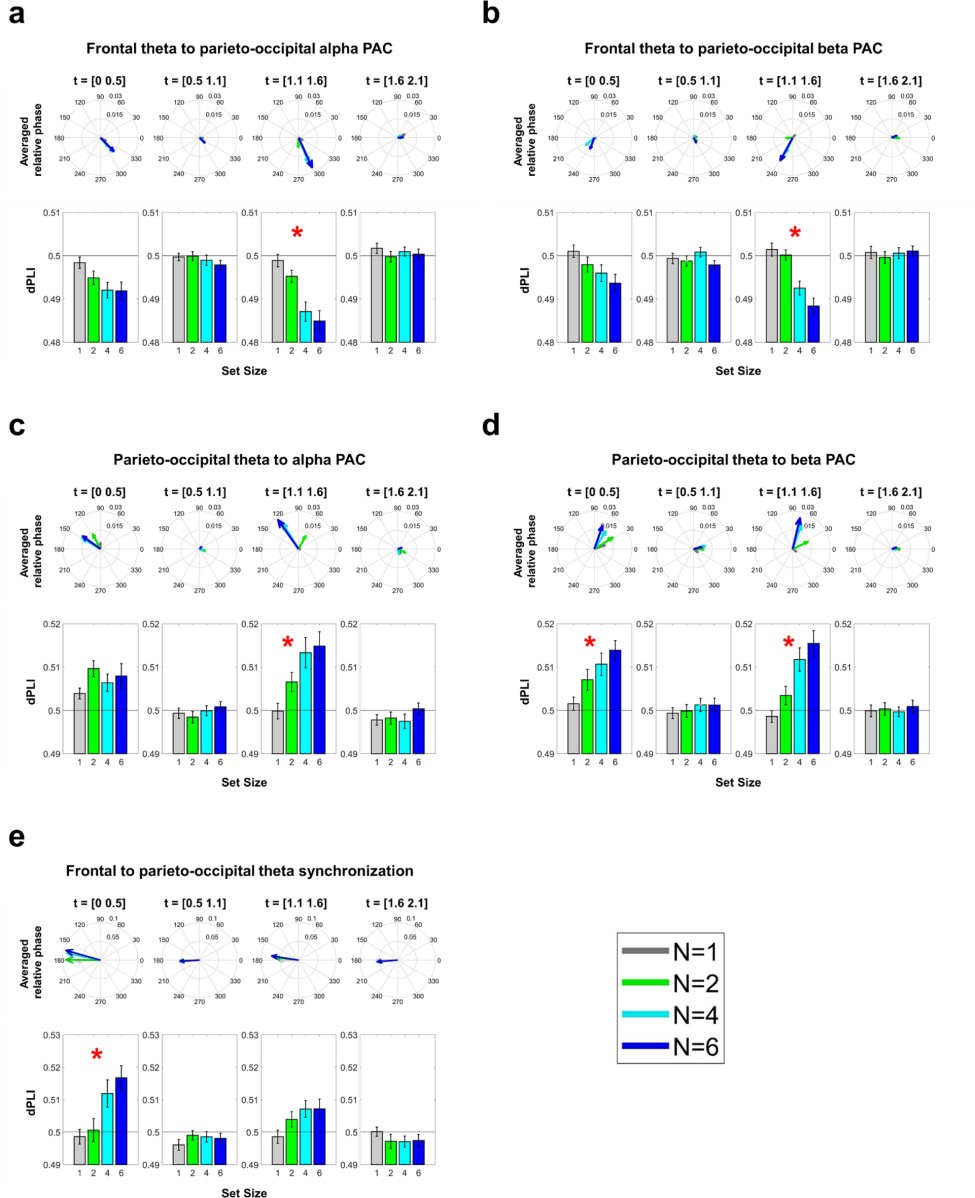
Load effects of inter- and intra-regional PAC between theta and alpha/beta AMs. Vectors denoted the group average of relative phase for each set size. A repeated-measures ANOVA was applied to each box plot to evaluate the load effect. Red asterisks indicated significant load effects after FDR correction. (**a**), (**b**) The inter-regional PACs between frontal theta phase and parieto-occipital alpha/beta AMs. (**c**), (**d**) The intra-regional PACs between parietal-occipital theta phase and alpha/beta AMs. (**e**) The dPLI between frontal and parieto-occipital theta oscillations. Significant load effects were observed during the onset of sample and probe arrays. For all significant cells, the dPLI deviated more from 0.5 as set size increased. For both the alpha and beta AMs, the direction of inter-regional PAC was the opposite when compared with intra-regional PAC. The phase lag between frontal and parieto-occipital theta phases was close to 180 degrees, suggesting a single source of theta activity.

dPLIs between theta-, alpha-, and beta-band oscillations (i.e., IMFs 6, 5, and 4, respectively) were calculated across all electrode pairs, then were averaged according to set-size conditions, frequency combination, and the locations of electrode pairs. Since the theta power increased but the alpha/beta power decreased in response to the task stimuli, a 180-degree offset should be considered when inferring the phase leading-lagging relationship from the cross-frequency dPLI. We found significant load effects in the first 0.5-s time window of the delay and probe periods (Fig. 8). The cross-frequency dPLI between frontal theta phase and parieto-occipital alpha/beta AM decreased from 0.5 to even smaller values during the onset of the probe array (*F*_*(3,64*)_ = 14.7782, *p* = 5.29 × 10^–6^ and *F*_*(3,64*)_ = 17.5511, *p* = 8.38 × 10^–7^; Fig. 9a,b). For cross-frequency connectivity within the parieto-occipital region, the dPLI between theta phase and alpha/beta AM increased from 0.5 to even larger values as set size increased during the onset of the sample and probe arrays (*F*_*(3,64*)_ = 6.4161, *p* = 0.0029 and *F*_*(3,64*)_ = 17.9176, *p* = 6.62 × 10^–7^ for theta-beta PAC; *F*_*(3,64*)_ = 7.8448, *p* = 0.0009 for theta-alpha PAC; Fig. 9c,d). Finally, the dPLI between frontal and parieto-occipital theta phases increased from 0.5 to larger values as the set size increased (*F*_*(3,64*)_ = 22.7148, *p* = 3.51 × 10^–8^; Fig. 9e).

In summary, the frontal theta phase preceded the phase of frequency-matched parieto-occipital alpha/beta AM under high WM loads, whereas the parieto-occipital theta phase followed the phase of frequency-matched alpha/beta AM within the same region. The frontal theta phase also preceded the parieto-occipital theta phase during the onset of the sample array. Note that the phase offsets between inter- and intra-regional dPLI were closed to 180 degrees under high WM load (e.g., N = 6), and the offsets between frontal and parieto-occipital theta phase were also closed (despite not equal) to 180 degrees in the same condition. Therefore, the pattern of intra-regional dPLI could arise from the field-spreading of the frontal theta wave.

We performed GLM analyses between the individual differences in WM precision and PAC, but neither intra- nor inter-regional PACs predicted individual WM precision. Finally, the degree of PAC did not predict the subsequent suppression of the parieto-occipital alpha/beta AM. These results indicated that PAC and AM are independent measurements in WM processing.

## Discussion

This study investigated the electrophysiological bases of AM and PAC in WM processing. The main result showed that stronger load-dependent alpha/beta power suppression in parieto-occipital electrodes was associated with better WM precision. Furthermore, the observed sustained alpha/beta power suppression could be separated into continuous low-frequency (< 2 Hz) and 4–12 Hz AMF components. The low-frequency component extended throughout the task procedure, whereas the 4–12 Hz AMF component was observed only after processing the sample and probe stimuli. When correlating individual WM precision to alpha/beta AMs, we found negative correlations for the tonic alpha/beta power suppression (AMF < 1 Hz) throughout the task procedure. On the other hand, higher AMFs of alpha/beta power suppression only showed significant correlations after the onset of the probe array.

Connectivity analysis with cross-frequency dPLI showed a load-dependent increment of theta-alpha/beta PAC between the frontal and parieto-occipital regions and within the parieto-occipital region itself during the onsets of the sample and probe arrays. Therefore, the theta-AMF component of parieto-occipital alpha/beta AM was likely driven by frontal and parieto-occipital theta activity. The overall pattern of results suggests that WM precision, like capacity measurements, is supported by frontoparietal oscillatory activities^21,57,58^.

### Alpha/beta power suppression reflects the ability to access feature information in WM

Our findings in parieto-occipital alpha/beta activity are consistent with previous reports showing that load-dependent alpha/beta suppression predicts individual differences in WM capacity^6,7,56,59^. In memory formation and retrieval, reducing alpha/beta power facilitates information-processing ability in task-relevant sensory areas^60^. Therefore, the precision of memorized information is associated with the ability to maintain alpha/beta power suppression in areas storing stimulus-specific information ^16^. Since the κ value measured the quality of WM representation^38^, our results suggest that maintaining fine-grained perceptual information in WM requires engaging the parieto-occipital region^14^. This argument is consistent with studies using multivariate regression methods, which have shown that non-spatial feature information of visual WM could be decoded from bilateral posterior alpha/beta activities^8,61,62^. Similar results have also been reported in fMRI studies, in which the stimulus information can be decoded from the pattern of BOLD responses in visual areas^63–65^. In addition, the same pattern of results can extend to other modalities, e.g., the load-dependent alpha/beta power suppression could either be observed within the visual or somatosensory area, depending on which modality was prompted for a response in an intermodal WM task^5^.

The alpha/beta power suppression pattern indicates two possible roles in WM processing. First, it is the neural substrate of the number of items held in memory^7,66^. Second, it reflects a necessary mechanism that keeps the target information activated, i.e., keeping relevant information in the focus of attention^67,68^. The first argument suggests that the alpha/beta power suppression should be reduced after presenting the retro cue since irrelevant information should be deactivated to prevent interference. The second argument does not assume the release of suppression after retro cue presentation. Our result finds alpha/beta power suppression does not reduce after presenting the retro-cue. The result is consistent with a previous report using lateralized cueing paradigm^53^. In that study, only the alpha power representing the cued location was suppressed, but the alpha power representing irrelevant item locations did not increase. One recent study has shown that the parieto-occipital alpha power suppression not only scales with WM load but also to the spatial extent between items and the target-distractor similarity^69^. Therefore, the alpha/beta power suppression observed in our study is better explained as the difficulty of maintaining the target information in the focus of attention.

By decomposing parieto-occipital alpha/beta AMs into different AMFs, we found differences between the trend (< 1 Hz) and higher AMF components (4–12 Hz) in their correlation to κ values. Specifically, the trend component showed a sustained negative correlation throughout the maintenance period, whereas the higher AMF components only showed a significant correlation after the onset of the probe array. These results might imply different cognitive factors contributing to the maintenance of WM information. The low-frequency component reflected tonic psychophysiological states such as alertness and sustained attention^70^. In contrast, the higher AMF components reflected within-trial target select processes^24^. The two levels of modulation are further evidence supporting parieto-occipital alpha/beta power as the access rather than the neural substrate of memorized visual information.

The *CF-AMF* structure of the higher AMF components shifted from alpha AM in the early (t = [1.1, 1.6] s) and to beta AM in the later (t = [1.6, 2.1] s) time windows of the probe period. One recent study has shown that the load-dependent increment of the parietal alpha/beta oscillation speed predicts individual WM capacity^71^. Therefore, the beta AM correlate observed might indicate a more efficient target selection for high-precision participants.

### Relationship between the frontal theta power and WM precision

Frontal midline theta activity has been considered the oscillatory substrate for cognitive control or cognitive efforts in completing the task^72,73^. Prior studies have associated the increment of frontal midline theta power with increasing memory load^5,22,74^, increasing task difficulty^75^, or as an index of successful WM manipulation^76^. The increment of frontal theta power is also associated with better performance in WM tasks^77,78^. Our results showed a positive correlation between frontal theta power and WM precision. However, frontal theta power did not scale with WM loads (Figs. S3 and 7a). The seeming contradiction of load effects could be explained as the following. First, frontal theta power increment is more sensitive in maintaining temporal order information than visual item information^79,80^. The effect size of frontal theta power increment could be smaller for concurrent visuospatial WM tasks. Second, other factors such as individual WM capacity and acute stress could interact with the frontal theta load effect so that frontal theta power peaks at some critical load and then decreases at higher loads^77,78^. One recent intracranial EEG study also failed to replicate the load-dependent theta power increment in the anterior cingulate cortex, which was considered the primary source of frontal midline theta activity^81^. The study argued that the hippocampus indeed contributed a portion of the frontal midline activity observed from the scalp.

### Frontoparietal cross-frequency connectivity indicates the top-down modulation of memorized contents

Our results show the load-dependent modulation of cross-frequency dPLI between frontal theta phase and frequency-matched parieto-occipital alpha/beta AMs (Fig. 9). The degree of dPLI increases as WM load increases, and such long-range modulation is only transiently involved during the processing of probe stimuli. The frontal theta phase precedes the phase of parieto-occipital alpha/beta AM. Therefore, the inter-regional PAC observed can be associated with the selection of visual information in WM^82^. Load effects for cross-frequency dPLI within the parieto-occipital cluster are also observed. However, the parieto-occipital theta phase falls behind the phase of alpha/beta AM. The result suggests that the state of alpha/beta AM may guide the upcoming sensory processing, thus forming a reciprocal interaction between the goal at the moment and perceptual inputs.

Previous studies have suggested that the prefrontal cortex serves as the central executive module during WM processing^1,83^. In particular, the prefrontal cortex exerts control through low-frequency oscillations around the theta-band, coordinating posterior areas that store WM representations^12,13,84–86^. Studies with spatial retro-cueing consistently reported stronger alpha power suppression in the contralateral sites^7,53,54^. Such alpha lateralization was transient following the retro-cue^87^, and the degree of lateralization scaled with task difficulty^88^. The transient lateralization of posterior alpha power was associated with the power of frontal low-frequency oscillations^62,87^. Our result is consistent with these findings.

Recent studies have proposed two mechanisms for frontal theta activity exerting control over posterior regions during memory. For the direct mechanism, the frontal theta activity determines the timing of long-range communication between the central executive and sensory areas^89^. The direct mechanism does not require long-range theta coherence as well as local PAC. On the other hand, the indirect mechanism assumes that the frontal region exerts control through long-range theta coherence. The activated representations are modulated by local PAC in the posterior region^19,90–92^. The direct modulation better explains our result since the long-range theta coherence is not necessary for frontoparietal theta to alpha/beta PAC.

### HHSA reveals the relationship between oscillatory power variations and PAC

While conventional PAC methods calculate the distribution of the power of a high-frequency oscillation to the phase of a low-frequency oscillation, the current study isolates the estimation of cross-frequency coupling in two steps: first, the amplitude of an oscillation was decomposed into different AMs with HHSA; second, PAC was evaluated by cross-frequency dPLI between the slow oscillation and the scale-matched AM of the fast oscillation^21,34^. We have shown that WM load manipulation modulates theta-alpha and theta-beta PACs during the processing of probe stimuli, followed by a corresponding change in alpha/beta power suppression. However, only alpha/beta AM predicts individual WM precision. The results imply PAC and AM have distinct meanings in WM processing. Theta-alpha/beta PAC reflects the mechanism of top-down selection over WM representations^12^, whereas alpha/beta AM reflects the fidelity of target information after selection.

### The possible roles of alpha/beta power suppression in the WM subprocesses

The 100 ms stimulus presentation time in this study falls short of the duration required for full encoding of visual stimuli into WM^93^. Therefore, the identified load effects on parieto-occipital alpha/beta power suppression may be resulted from unfinished encoding process, rather than the active maintenance of WM representation.

Parieto-occipital alpha/beta power suppression has been frequently documented in memory studies involving simple features such as color and orientation^6,7,56,94^. This sustained suppression is observed during more extended encoding and maintenance periods. For instance, Proskovec et al. (2019) utilized 1.5 s for encoding and 2.5 s for maintenance in their research, and alpha power suppression was observed across the trial sequence. Their findings suggest a maintenance role for the sustained parieto-occipital alpha/beta power suppression. On the other hand, when dealing with information that can be transformed into other modalities, such as shapes or letters, parieto-occipital alpha/beta power decreases during encoding and increases during maintenance^18,95^. These studies suggest that task-relevant information can be transferred to other brain areas for sustained maintenance. In this context, the observed parieto-occipital alpha/beta power suppression indicates essential visual information processing for memory encoding.

Taken together, it is evident that the occurrence of parieto-occipital alpha/beta power suppression during maintenance is contingent upon the nature of the tested features. If the alpha/beta suppression is solely a consequence of visual information encoding, it would not persist during the maintenance period for tasks involving simple features with sufficient encoding duration. Therefore, we conclude that the alpha/beta power suppression observed in this study is related to the active maintenance of memorized information. Nevertheless, it is important to note that this does not preclude its potential involving in the encoding process. Further investigation is required to elucidate the distinct contributions of encoding and maintenance to alpha power suppression in WM tasks of similar design.

## Conclusion

Our results demonstrate that individual differences in parieto-occipital alpha/beta power suppression during WM maintenance predict their recall precision. The alpha/beta power suppression can be decomposed into different AMF components. The faster (4–12 Hz) and the slower (< 2 Hz) AMF components represent within- and across-trial attentional mechanisms that are crucial for maintaining the activated WM information. Enhanced theta-alpha/beta frontoparietal PAC under high WM load suggests an explicit mechanism for inter-regional communication. When a target-defining is presented, the frontal theta wave modulates parieto-occipital alpha/beta AM with PAC, and the subsequent alpha/beta AM variation determines the recall precision.

## Limitations

This study finds that frontoparietal theta-alpha/beta PAC increases with increasing WM load, and individual differences in WM precision are associated with parieto-occipital alpha/beta AM around the theta-band (4–12 Hz) AMF. Nevertheless, since AM summarizes multiplicative interactions from all possible sources, the reported results could also arise from the task design itself. More experiments are necessary to verify whether the observed alpha/beta AM correlates depending on the task design. For instance, if the same periodic EEG activities modulate WM maintenance, the same AM correlates are expected under different delay periods. From the pattern of results, we inferred that frontal theta oscillation served as the executive control, whereas parieto-occipital alpha/beta AM reflected attentional selections that are required for maintaining activated WM representations. Both arguments need to be examined with direct testing, such as manipulating different levels of cognitive control under the same stimulus display. Finally, the paradigm was modified from the standard WM precision task^37^, in which a retro cue was presented the probe participants’ memory one seconds before the response stage. The comparable results in the *CF-t* spectrum might indicate the generalizability of HHSA results in similar tasks.

### Supplementary Information

Supplementary Information.

## Data Availability

The data and necessary codes supporting the findings of this study are available in the OSF repository (http://osf.io/243ps).

## Abbreviations

AM: Amplitude modulation
AMF: Amplitude-modulation frequency
CF: Carrier frequency
CFC: Cross-frequency coupling
dPLI: Directed phase-lag index
DQ: Direct quadrature
EMD: Empirical mode decomposition
FDR: False-discovery rate
FO: Fast oscillation
GLM: General linear model
HHCFPC: Holo-Hilbert cross-frequency phase clustering
HHS: Holo-Hilbert spectrum
HHSA: Holo-Hilbert spectral analysis
IMF: Intrinsic-mode function
PAC: Phase-amplitude coupling
SO: Slow oscillation
WM: Working memory
