# Supplementary material for: The association between working memory precision and the nonlinear dynamics of frontal and parieto-occipital EEG activity
Source: Sci Rep. 2023 Aug 31;13:14252. doi: 10.1038/s41598-023-41358-0 (PMC10471634; doi:10.1038/s41598-023-41358-0)
Supplement: Supplementary file 1 — Supplementary Information. [file 41598_2023_41358_MOESM1_ESM.docx]

# Supplementary Information

## Post-hoc Analysis for Mixture-Model Parameters

For behavioral results, recall errors were separated into three possible sources by fitting a mixture model in each set size condition (see Methods section in the main text). For the model, pU denoted the guess rate, pNT denoted the rate of misreporting a non-target item, the rate for correctly recalling the target was pT = 1-pU-pNT, and κ denoted the precision parameter (i.e., the reciprocal of standard deviation) for the error distribution under correct recalling. ANOVA analyses revealed significant load effects for all four parameters (Fig. 1c). For post hoc analysis, we compared set sizes 1 vs. 2, 2 vs. 4, and 4 vs. 6 for all parameters. The results were corrected with the Benjamini-Hochberg test for multiple-comparison correction (FDR was set at 0.05 for two-tailed tests). As set size increased, pT and κ decreased, whereas pU and pNT increased (Table S1). Among the four parameters, only κ showed significant differences for comparison between set sizes 1 and 2. pT, pU and **κ** were significant for comparison between set sizes 2 vs. 4 and 4 vs. 6, but pNT only showed marginal significances.

| **Table S1**.  Post hoc analysis for mixture model parameters | | | | |
| --- | --- | --- | --- | --- |
|  | **t-statistic (p value)** † | | | |
| **Contrast** | **κ** | **pT** | **pNT** | **pU** |
| **1 vs 2** | -2.728 (0.0103)* | -1.2182 (0.2321) | N/A†† | 0.0915 (0.9277) |
| **2 vs 4** | -4.7273 (4.38×10^-5^)* | -7.4262 (1.89×10^-8^)** | 2.3583 (0.0246) | 5.7989 (1.94×10^-6^)** |
| **4 vs 6** | -3.8889 (4.78×10^-4^)* | -4.6453 (5.55×10^-5^)* | 2.4185 (0.0215) | 2.581 (0.0146)* |
| *Note*. A Benjamini-Hochberg procedure controls the FDR at .05 level.  * indicates the false discovery rate (FDR) is smaller than 0.025; ** indicates FDR < 0.005. | | | | |
| † The degrees of freedom are 32 for all comparisons. | | | | |
| †† pNT is undefined for the set-size 1 condition. | | | | |

## Comparison of AM and PAC

Here we use a simulation to address the difference between AM and PAC (Fig. S1) [1]. The simulation compares the spectral representations of AM and PAC signals as well as their constituent waves. The spectral representations of the signals are generated with HHSA [2]. In this simulation, the high-frequency component of the AM and the PAC signals are modulated in the same frequency, but only the PAC signal contains the frequency-matched slower components (Fig. S1a). However, the standard PAC method [3] only extracts the AM energy in the PAC waveform, despite both the AM and PAC waveforms both contain AM energy in the same modulating frequency (Figs. S1b and S1c).


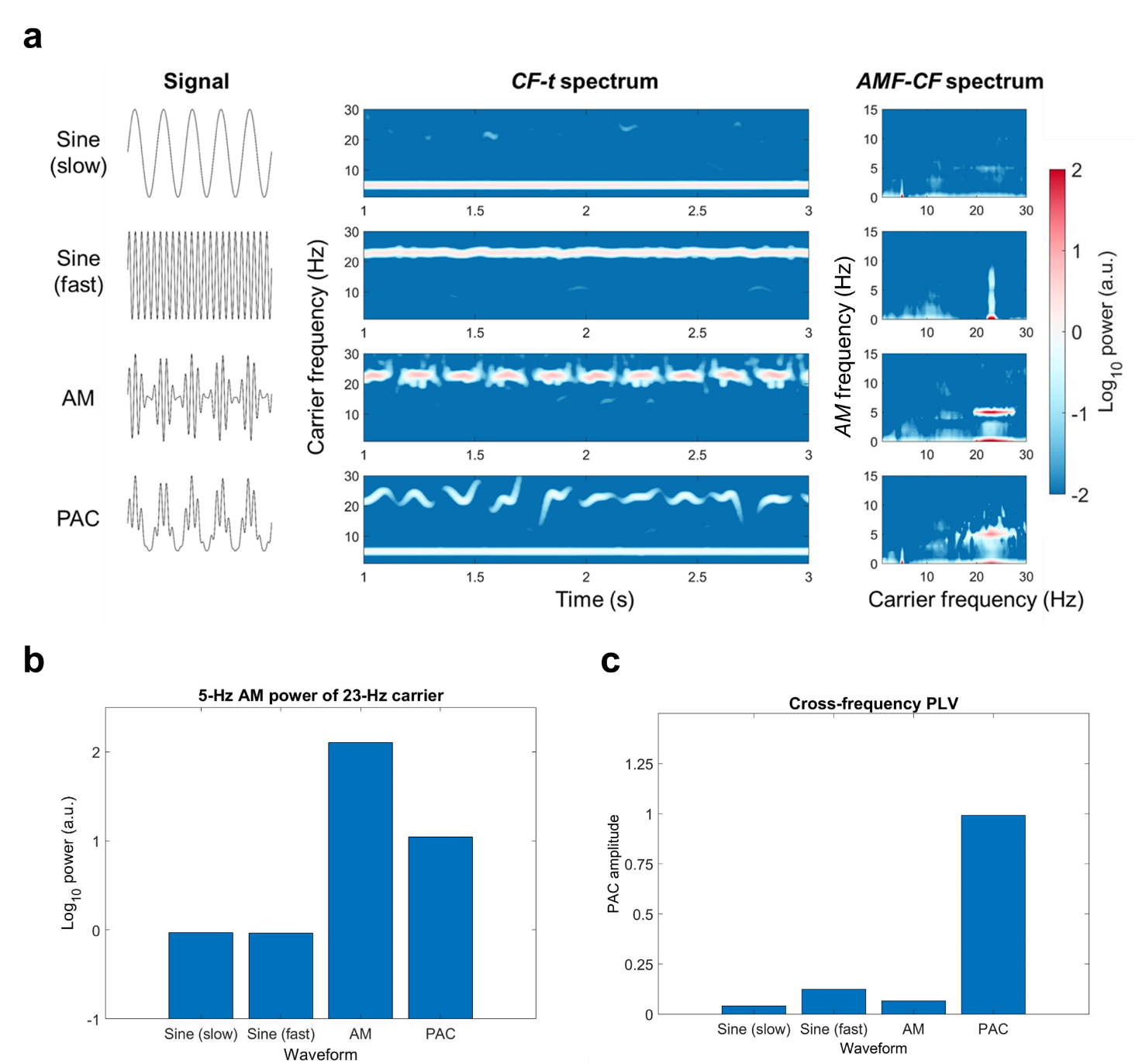


**Figure S1. Comparison of PAC amplitude and AM power in different waveforms.** (a) Simulated waveforms and their spectral representations. The waveforms are decomposed by HHSA. (*Left column*) The tested waveforms are a 5-Hz sine wave, a 23-Hz sine wave, a 23-Hz sine wave with 5-Hz AM, and a PAC waveform composed of a 5-Hz sine wave and a 23-Hz sine wave with 5-Hz AM. (*Middle column*) The time-frequency representation of the simulated waveforms. The intermittent rhythmic patterns for the AM and PAC waves indicate the existence of AM for the 23-Hz carrier. (*Right column*) The *AMF-CF* representation of the simulated waveforms. The horizontal axis denotes carrier frequency, and the vertical axis denotes the AM frequency. The energy of the fast and the slow sine waves are concentrated in their corresponding carrier frequencies without AM (i.e., 0 Hz). For the AM wave, the energy is concentrated at 23-Hz CF, both at the DC level and 5-Hz AMF. An additional 5-Hz carrier-frequency component can be observed at the DC level for the PAC wave. (b) The 5-Hz AM power at 23-Hz carrier frequency with for the four waveforms tested. (c) PAC amplitudes between 5-Hz and 23-Hz CF components. Only the PAC wave shows a large PAC amplitude.

## Amplitude Variations from IMFs 4 to 7

In the preliminary analysis, we tested the effect of WM load manipulation to IMF powers averaged across all electrodes. The single item condition served as the baseline and was subtracted from conditions of set sizes 2, 4, and 6 for comparison. For each time point in each IMF, a repeated-measures ANOVA was tested for set sizes 2, 4, and 6. The results were corrected for multiple comparisons across time and IMF by a cluster-based permutation test, where both the cluster-forming threshold and significant levels were set at *p* = 0.05. Significant clusters for the load effect were observed in IMFs 4 to 7 among all 10 IMFs tested (Fig. S2). Negative clusters were observed in both the delay and probe periods for IMF4 (11.8-23.6 Hz, beta band), following the evoked responses for the onsets of sample and probe arrays. For IMF5 (6.4-11.8 Hz, alpha band), a positive cluster could be observed during the onset of the sample array, and negative clusters were observed later in both the delay and probe periods. IMF6 (3.1-6.4 Hz, theta band) showed positive effects following the onset of the delay and probe arrays. Finally, IMFs 6 and 7 (1.5-3.1 Hz, delta band) both showed negative load effects in the last 0.4-s time window of the probe period. The overall pattern of results indicated two mechanisms modulating EEG activity in a load-dependent manner: a sustained power suppression in alpha/beta frequencies during WM maintenance period, which was superimposed by transient broad-band evoked responses following the onsets of the delay and probe arrays.


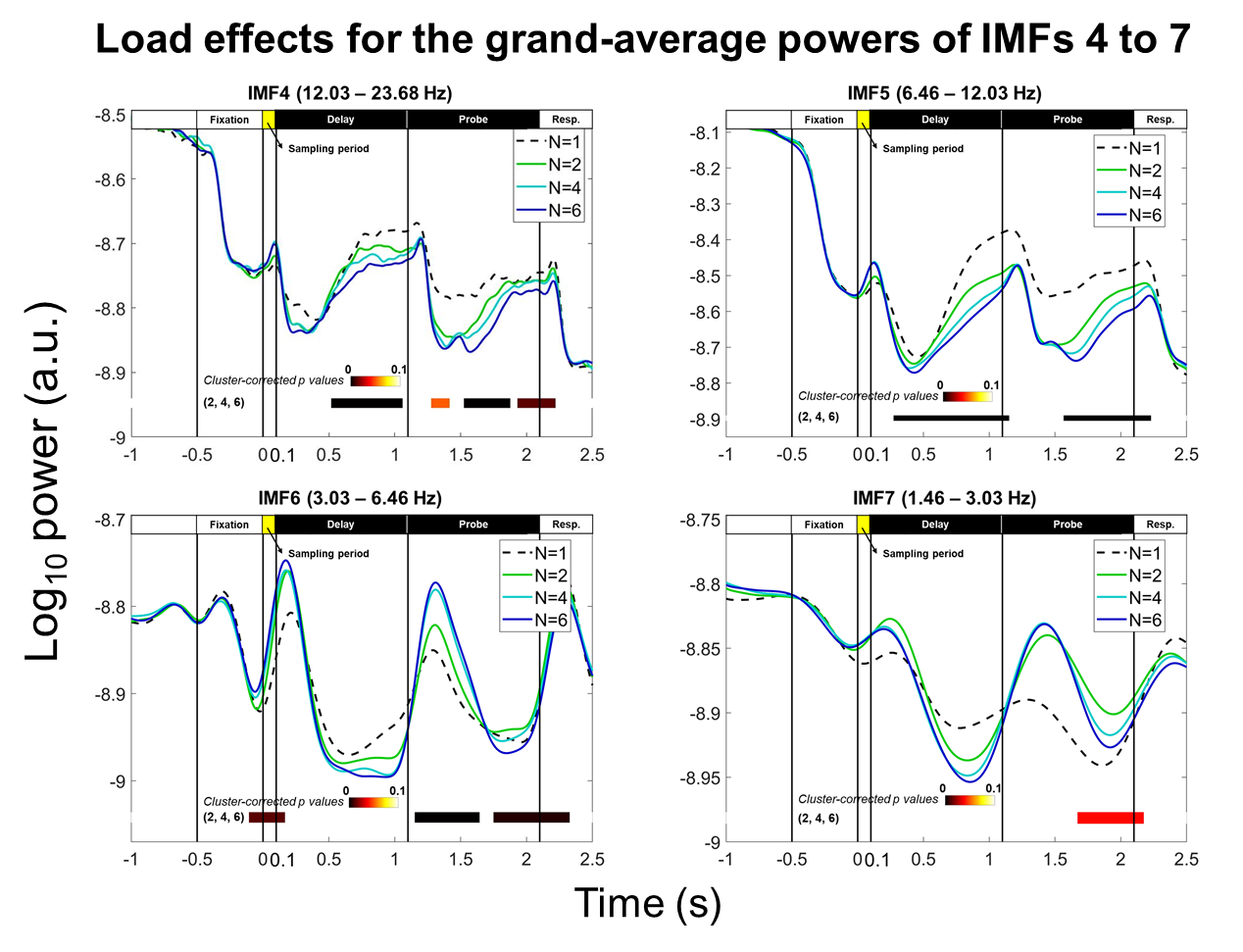


**Figure S2. Load-dependent modulation of powers in IMFs 4 to 7.** A repeated-measure ANOVA between set sizes 2, 4, and 6 was performed for each time point and each IMF with the single-item condition served as the baseline for comparison. The results of ANOVA were corrected by a cluster-based permutation correction for 5000 iterations. The color bar denoted cluster-corrected *p*-value for each cluster. (*upper panel*) IMFs 4 and 5 showed similar negative load effects after processing the sample and probe stimuli, as indicated by the transient power increment following the onset of the sample and probe arrays. IMF 5 also showed a transient positive load effect following the onset of the sample array. (*lower panel*) IMF 6 showed transient positive effects following the onset of the sample and probe arrays, and both IMFs 6 and 7 showed negative load effects in the last 0.4-s time window of the probe period.

## Load Effects for the CF-t spectrum in the Frontal Region

The *CF-t* spectra of the mid-frontal electrodes (AF3/4, F1/2/3/4, FC1/2/3/4, Fz, and FCz) was averaged for each set size. The single-item condition served as the baseline for comparison. A repeated-measure ANOVA for set sizes 2, 4, and 6 (subtracted from the single item condition) was tested at each spectral point to test the load effect. A cluster-based permutation procedure of 5000 iterations was applied for multiple corrections. Both cluster-forming threshold and significance level were set at *p* = 0.05. As illustrated in Fig. S3, no significant cluster was identified.


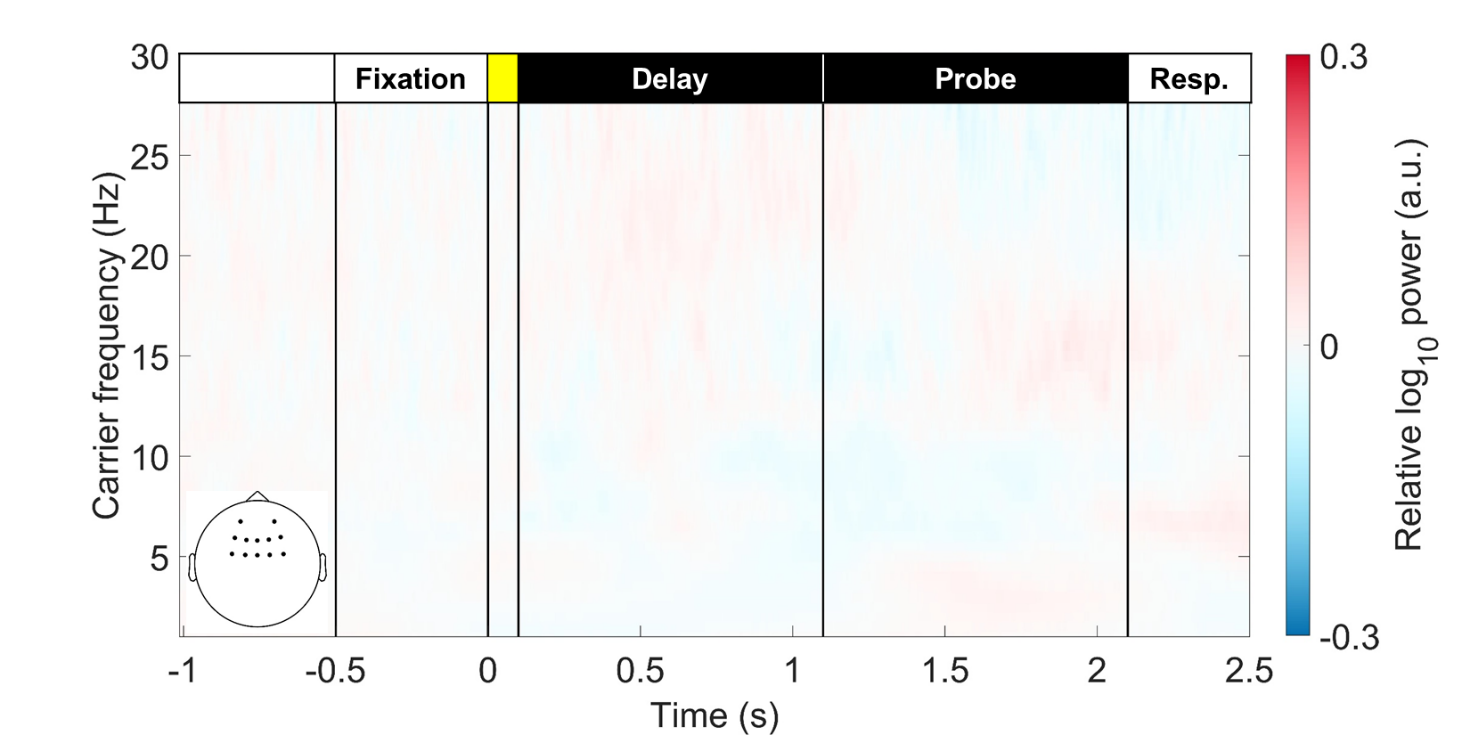


**Figure S3. The *CF-t* representation of load effects for frontal EEG power.** The figure illustrated the averaged *CF-t* spectrum in the frontal area for set sizes 2, 4, and 6, contrasting with the single-item condition. No significant load effect was detected throughout the WM retention period after cluster-based permutation correction with 5000 iterations. The cluster-forming and significance thresholds were set at *p* = 0.05.

## Comparison of EMD and Wavelet Decomposition

We have repeated the same spectral analysis for the effect of load manipulation and the correlation to WM precision (**κ**) using the Morlet wavelet transforms with 6 cycles. The power was calculated from 1 to 30 Hz in 0.5 Hz steps. Since there is no AMF dimension in wavelet analysis, only *CF-t* spectra needed to be considered. A repeated-measures ANOVA for set sizes 2, 4, and 6 was applied to test the effect of load manipulation where the set-size 1 condition served as the baseline. The results were consistent for EMD- and wavelet-based spectra (Fig. S4). Results of correlation analyses were similar for the two decomposition methods, too. However, the sensitivity for the Morlet wavelet transform was smaller so that the positive correlation of frontal theta power failed to reach significancy (Fig. S5).


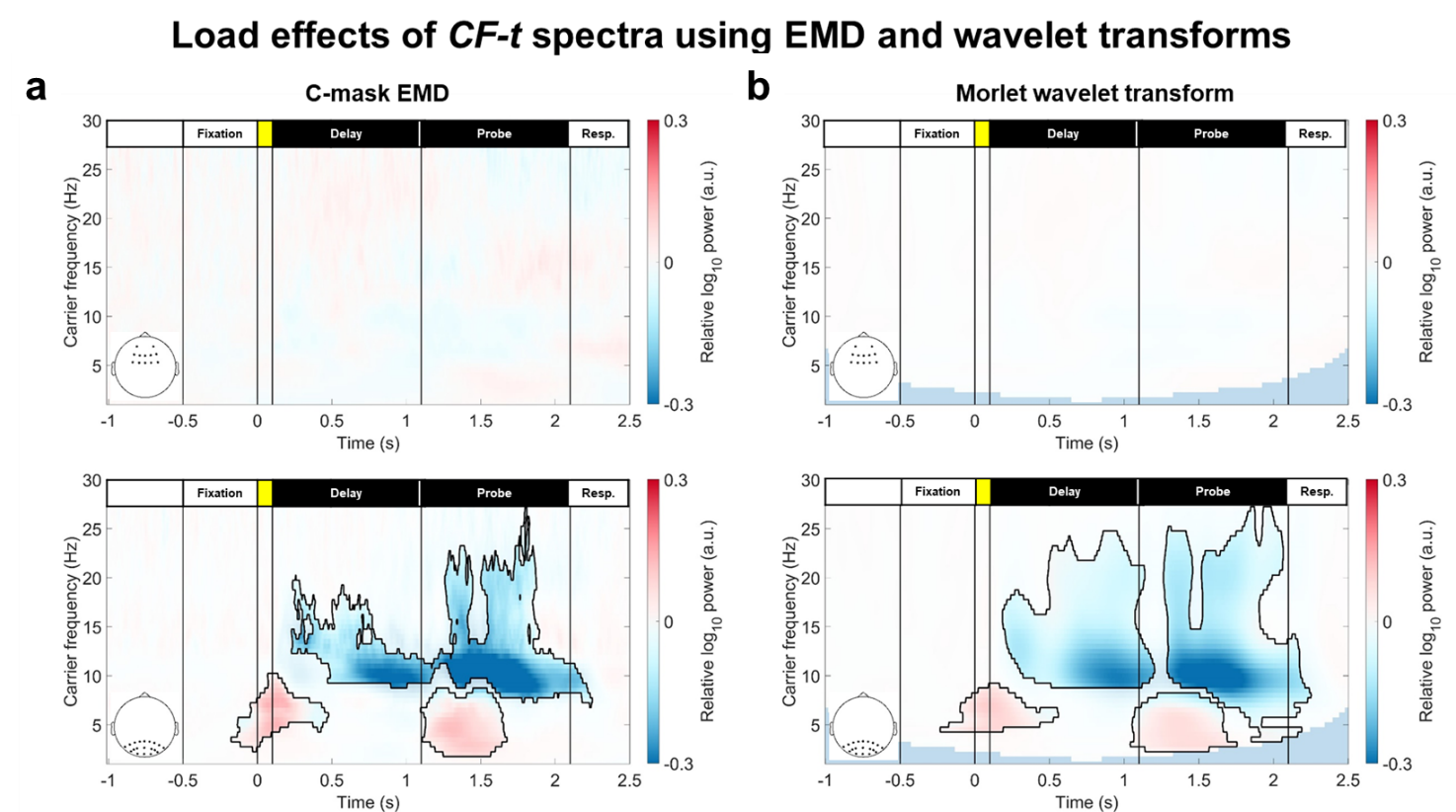


**Figure S4. Comparison of *CF-t* representations of load effect using C-mask EMD and the wavelet decomposition.** The effects of load manipulation were similar for C-mask EMD (a) and the wavelet-based *CF-t* spectra (b). No effect was observed in the frontal region. Positive theta-band clusters could be observed in the onset of the sample and probe arrays. Negative clusters in the alpha and beta bands were observed in the delay and probe periods, after the onsets of stimuli.


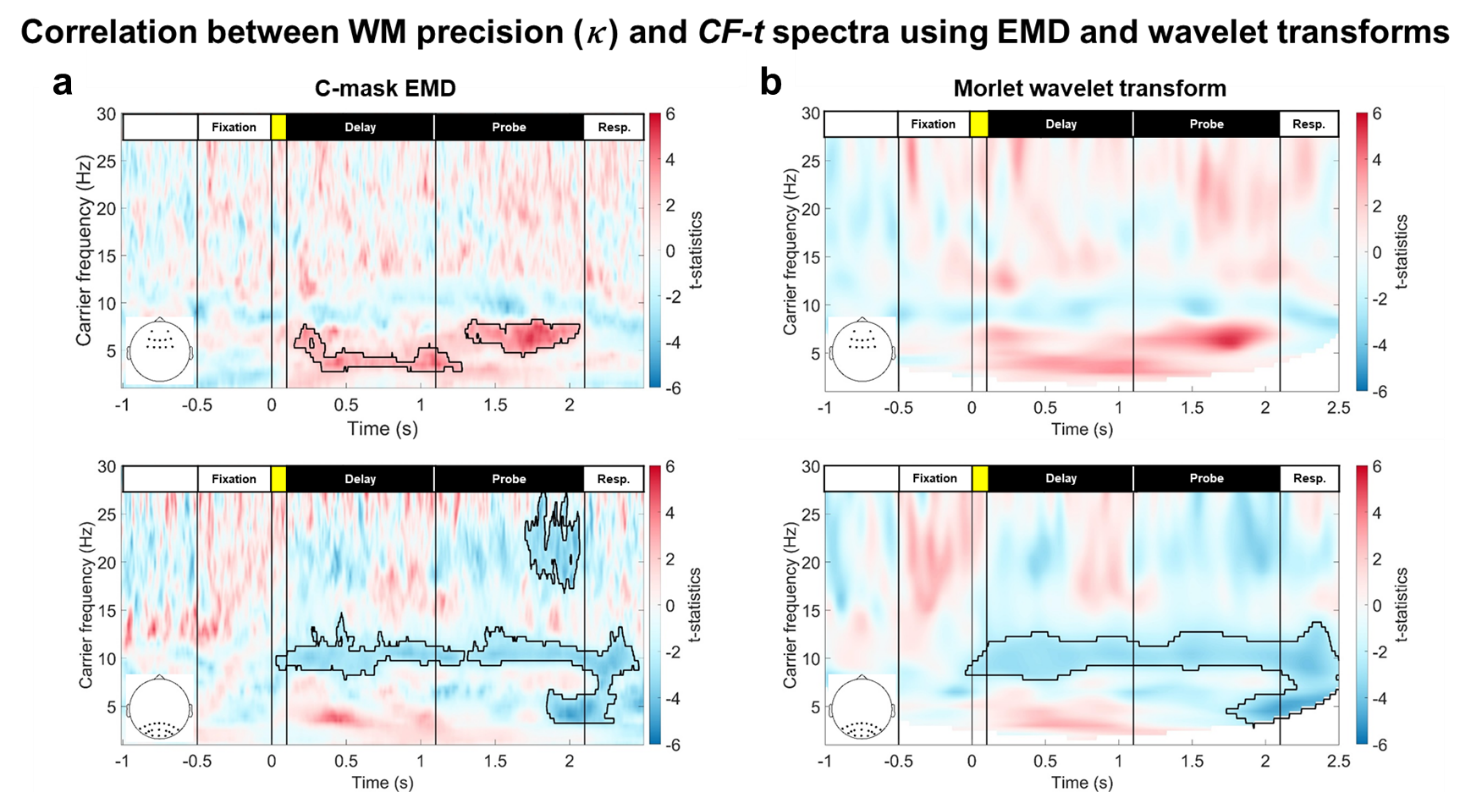


**Figure S5. Comparison of *CF-t* representations of WM precision using C-mask EMD and the wavelet decomposition**. The results were similar between *CF-t* spectra generated with (a) C-mask EMD and (b) the Morlet wavelet transform. However, the EMD-based spectra showed higher sensitivity in detecting correlations.

## Frequency Distribution of IMFs

It has been shown that EMD acts as a dyadic filter bank similar to wavelet decomposition in the same frequency ranges [4]. Each IMF represents oscillatory activity in different log-2 timescales. To demonstrate our data has the same behavior, we have added descriptive statistics regarding the between-participant variation of the frequency range for each IMF in the main text (Table 1). Details of each participant’s peak frequency and the lower bounds are summarized in Tables S2 and S3. The peak frequency and the boundaries for all IMFs of interest (i.e., IMFs 4, 5, and 6) are consistent across participants. The amplitude spectral density of IMFs 4, 5, and 6 are illustrated in Fig. S6.


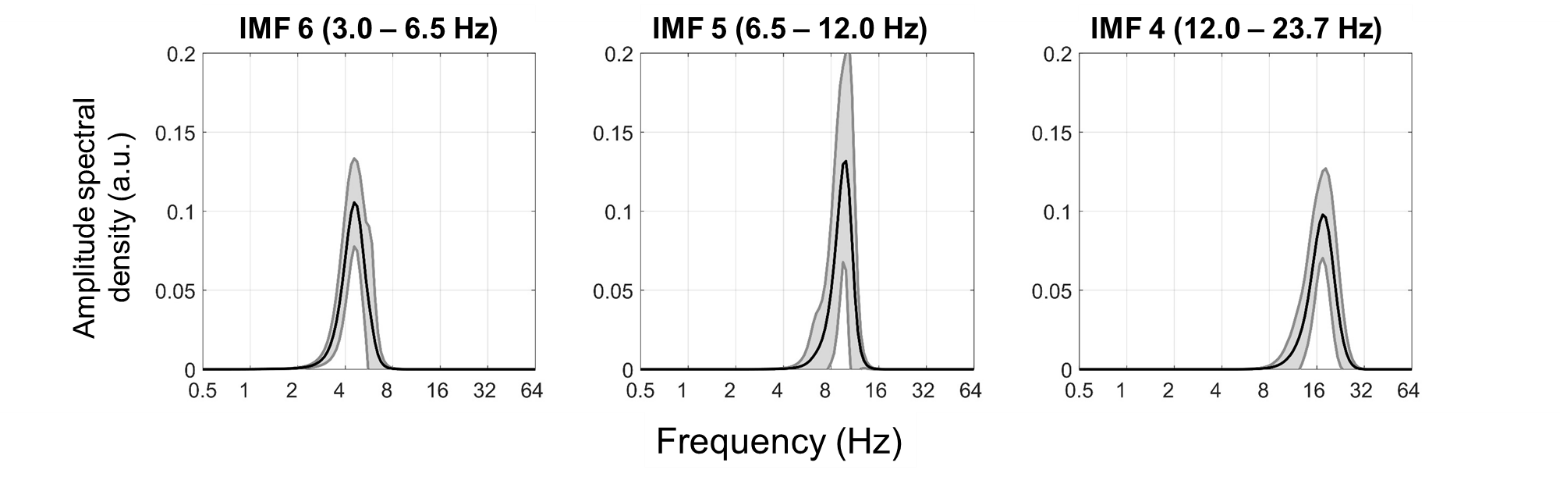


**Figure S6. Amplitude spectral density of IMFs 4, 5, and 6.** Black line denotes the mean and gray area denotes the 95% confidence interval across all participants. The bandwidth of IMFs 6, 5 and 4 lie within conventional theta-, alpha-, and beta-bands, respectively.

## Comparing Load Effects in Full and Induced HHS.

Given the brief 100 ms stimulus-presentation duration, the stimulus-evoked potential introduces a potential confounding factor to the primary outcomes. To address this concern, we mitigate this possibility through a parallel analysis of the load-effect using induced EEG activity. Induced activity is derived by subtracting the evoked spectrum from the original full spectrum. To calculate the evoked spectrum, we first calculate the event-related potential of each set size for each participant, then subtract the averaged signal to HHSA (Fig. S7a). The findings of the load-effect analysis demonstrate that the persistence of sustained alpha suppression in the induced activity, both in the *CF-t* (cluster-corrected *p* = 4*10^-4^) and *AMF-t* (cluster-corrected *p* = 2*10^-4^) spectra (Fig. S7b, right column). Conversely, the transient theta clusters following the sample and probe array onsets are absent in the induced spectrum analysis. In addition, an identified low-frequency component emerges during the probe period (cluster-corrected *p* = 1.1*10^-3^).


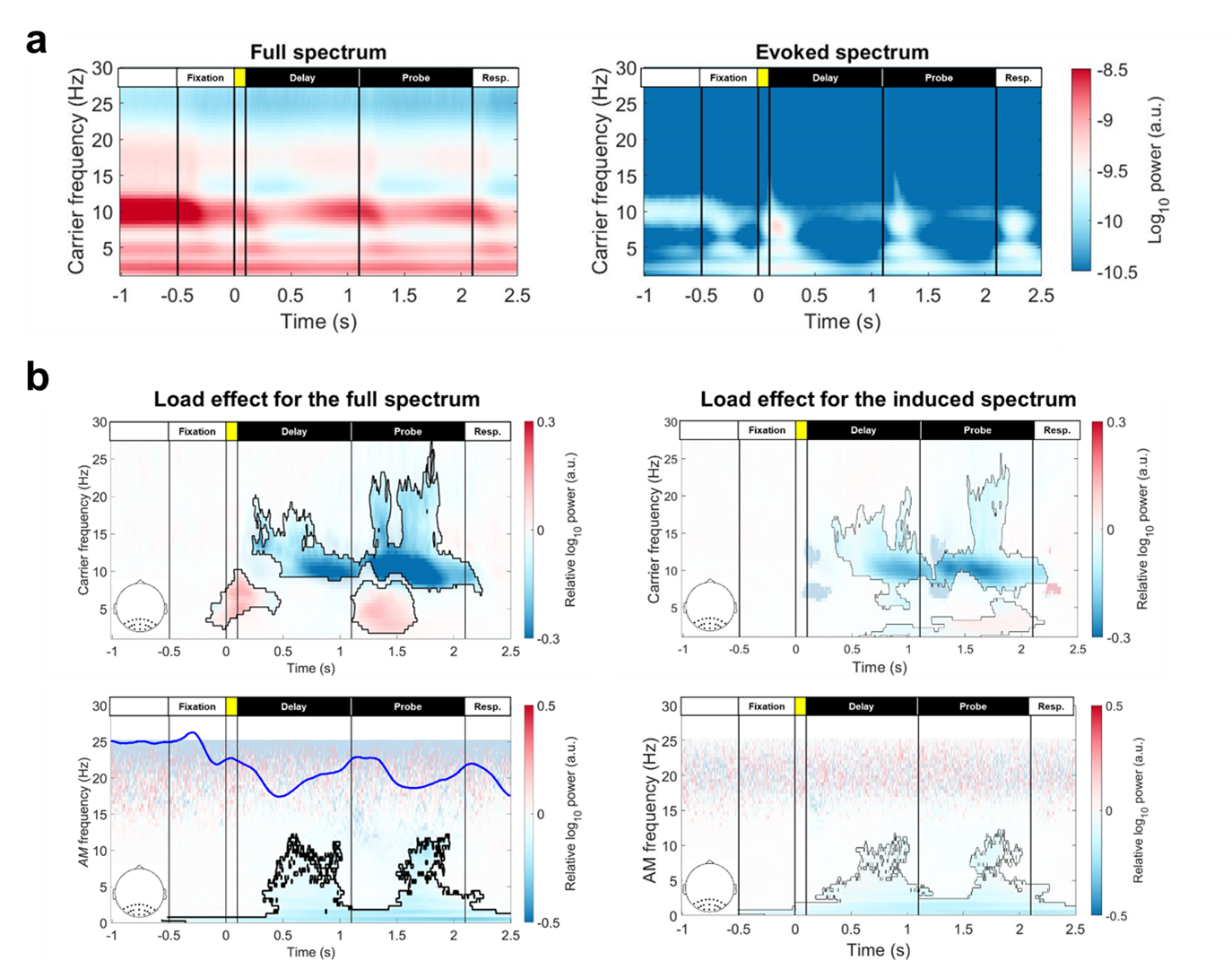


**Figure S7. Comparison of load effects in the full and induced power spectra.** (a) In the full activity, the *CF-t* spectrum showed sustained power across various frequency bands, whereas the *CF-t* spectrum in the evoked activity showed transient power increment following the onset of stimuli. (b) The left column represents the original results of load analysis, while the right column displays the results of load analysis for the induced activity. In the *CF-t* spectrum, the sustained suppression of alpha power is preserved (cluster-corrected *p* = 4*10^-4^), but the positive transient response in the low-frequency range disappears. In addition, a low-frequency component during the probe period is identified (cluster-corrected *p* = 1.1*10^-3^). The result in the *AMF-t* spectrum for the induced activity is consistent with the original result (cluster-corrected *p* = 2*10^-4^).

| **Table S2**  *The Peak Frequency of each IMF for All Participants* | | | | | | | | | | |
| --- | --- | --- | --- | --- | --- | --- | --- | --- | --- | --- |
| **Participant** | **IMF 10** | **IMF 9** | **IMF 8** | **IMF 7** | **IMF 6** | **IMF 5** | **IMF 4** | **IMF 3** | **IMF 2** | **IMF 1** |
| **1** | 0.22 | 0.59 | 1.19 | 2.38 | 4.97 | 10.83 | 19.87 | 36.44 | 69.79 | 133.67 |
| **2** | 0.23 | 0.55 | 1.09 | 2.18 | 4.56 | 9.11 | 17.45 | 34.90 | 64.00 | 128.00 |
| **3** | 0.24 | 0.57 | 1.09 | 2.28 | 4.76 | 10.37 | 16.71 | 33.42 | 64.00 | 128.00 |
| **4** | 0.24 | 0.55 | 1.04 | 2.18 | 4.36 | 8.72 | 16.71 | 33.42 | 64.00 | 122.57 |
| **5** | 0.23 | 0.57 | 1.04 | 2.18 | 4.36 | 10.37 | 16.71 | 33.42 | 64.00 | 122.57 |
| **6** | 0.23 | 0.59 | 1.09 | 2.38 | 4.97 | 9.51 | 17.45 | 34.90 | 69.79 | 133.67 |
| **7** | 0.23 | 0.57 | 1.00 | 2.09 | 4.56 | 9.11 | 17.45 | 32.00 | 64.00 | 122.57 |
| **8** | 0.23 | 0.55 | 1.00 | 2.28 | 4.56 | 9.51 | 18.22 | 33.42 | 66.83 | 128.00 |
| **9** | 0.23 | 0.57 | 1.09 | 2.18 | 4.56 | 9.11 | 18.22 | 34.90 | 64.00 | 128.00 |
| **10** | 0.24 | 0.57 | 1.09 | 2.18 | 4.56 | 9.93 | 17.45 | 32.00 | 64.00 | 122.57 |
| **11** | 0.23 | 0.57 | 1.14 | 2.38 | 4.97 | 10.37 | 19.03 | 36.44 | 72.88 | 139.58 |
| **12** | 0.24 | 0.57 | 1.09 | 2.18 | 4.36 | 9.51 | 16.71 | 33.42 | 64.00 | 122.57 |
| **13** | 0.23 | 0.59 | 1.09 | 2.28 | 4.76 | 9.93 | 18.22 | 36.44 | 69.79 | 133.67 |
| **14** | 0.23 | 0.55 | 1.04 | 2.28 | 4.56 | 9.93 | 17.45 | 33.42 | 61.29 | 117.38 |
| **15** | 0.22 | 0.59 | 1.04 | 2.28 | 4.56 | 10.37 | 17.45 | 34.90 | 66.83 | 128.00 |
| **16** | 0.23 | 0.65 | 1.00 | 2.28 | 4.76 | 10.37 | 18.22 | 34.90 | 66.83 | 128.00 |
| **17** | 0.22 | 0.62 | 1.04 | 2.18 | 4.56 | 9.11 | 17.45 | 34.90 | 66.83 | 122.57 |
| **18** | 0.23 | 0.59 | 1.04 | 2.28 | 4.76 | 9.51 | 18.22 | 34.90 | 69.79 | 128.00 |
| **19** | 0.23 | 0.59 | 1.04 | 2.18 | 4.56 | 9.51 | 17.45 | 34.90 | 69.79 | 128.00 |
| **20** | 0.23 | 0.57 | 1.09 | 2.38 | 4.56 | 9.11 | 17.45 | 32.00 | 61.29 | 117.38 |
| **21** | 0.24 | 0.62 | 0.96 | 2.09 | 4.18 | 9.51 | 17.45 | 33.42 | 64.00 | 112.40 |
| **22** | 0.22 | 0.62 | 0.96 | 2.28 | 4.56 | 9.51 | 18.22 | 32.00 | 66.83 | 128.00 |
| **23** | 0.22 | 0.59 | 1.04 | 2.28 | 5.91 | 6.44 | 19.03 | 30.64 | 64.00 | 145.76 |
| **24** | 0.22 | 0.68 | 1.04 | 2.28 | 4.56 | 9.51 | 17.45 | 34.90 | 66.83 | 128.00 |
| **25** | 0.22 | 0.65 | 1.09 | 2.28 | 4.76 | 8.72 | 17.45 | 33.42 | 66.83 | 128.00 |
| **26** | 0.22 | 0.62 | 1.04 | 2.18 | 4.56 | 9.93 | 15.32 | 33.42 | 66.83 | 122.57 |
| **27** | 0.22 | 0.65 | 1.04 | 2.18 | 4.76 | 9.11 | 17.45 | 34.90 | 66.83 | 128.00 |
| **28** | 0.23 | 0.59 | 1.04 | 2.28 | 4.56 | 9.93 | 17.45 | 34.90 | 69.79 | 128.00 |
| **29** | 0.23 | 0.65 | 1.09 | 2.28 | 4.56 | 10.37 | 16.00 | 34.90 | 69.79 | 128.00 |
| **30** | 0.22 | 0.68 | 1.14 | 2.38 | 4.97 | 10.37 | 19.03 | 36.44 | 72.88 | 139.58 |
| **31** | 0.16 | 0.62 | 1.04 | 2.18 | 4.56 | 9.93 | 16.71 | 33.42 | 66.83 | 112.40 |
| **32** | 0.22 | 0.68 | 1.09 | 2.18 | 4.36 | 9.93 | 17.45 | 34.90 | 69.79 | 128.00 |
| **33** | 0.23 | 0.59 | 1.04 | 2.18 | 4.76 | 10.37 | 16.00 | 33.42 | 64.00 | 122.57 |

| **Table S3**  *The Lower-Bound Frequency of each IMF for All Participants* | | | | | | | | | |
| --- | --- | --- | --- | --- | --- | --- | --- | --- | --- |
| **Participant** | **IMF 9** | **IMF 8** | **IMF 7** | **IMF 6** | **IMF 5** | **IMF 4** | **IMF 3** | **IMF 2** | **IMF 1** |
| **1** | 0.40 | 0.77 | 1.61 | 3.22 | 7.34 | 12.88 | 25.77 | 51.54 | 98.70 |
| **2** | 0.39 | 0.71 | 1.48 | 2.95 | 6.44 | 11.81 | 23.63 | 47.26 | 90.51 |
| **3** | 0.39 | 0.74 | 1.48 | 3.08 | 7.03 | 12.34 | 23.63 | 47.26 | 86.67 |
| **4** | 0.39 | 0.71 | 1.41 | 2.95 | 5.91 | 11.81 | 22.63 | 45.25 | 86.67 |
| **5** | 0.39 | 0.71 | 1.41 | 2.95 | 6.17 | 12.34 | 22.63 | 43.34 | 86.67 |
| **6** | 0.40 | 0.74 | 1.48 | 3.22 | 6.44 | 11.81 | 23.63 | 47.26 | 90.51 |
| **7** | 0.39 | 0.71 | 1.41 | 2.95 | 6.44 | 11.31 | 22.63 | 45.25 | 86.67 |
| **8** | 0.39 | 0.71 | 1.41 | 2.95 | 6.17 | 11.81 | 23.63 | 47.26 | 90.51 |
| **9** | 0.34 | 0.74 | 1.48 | 2.95 | 6.17 | 12.34 | 23.63 | 47.26 | 90.51 |
| **10** | 0.39 | 0.74 | 1.48 | 2.95 | 6.17 | 11.81 | 22.63 | 45.25 | 86.67 |
| **11** | 0.40 | 0.77 | 1.61 | 3.22 | 7.34 | 12.34 | 25.77 | 51.54 | 98.70 |
| **12** | 0.39 | 0.71 | 1.41 | 2.95 | 6.17 | 10.83 | 22.63 | 45.25 | 86.67 |
| **13** | 0.39 | 0.77 | 1.48 | 3.08 | 6.44 | 12.34 | 24.68 | 49.35 | 94.52 |
| **14** | 0.39 | 0.71 | 1.48 | 2.95 | 6.17 | 11.81 | 22.63 | 45.25 | 83.00 |
| **15** | 0.42 | 0.74 | 1.41 | 2.95 | 6.44 | 12.34 | 23.63 | 47.26 | 90.51 |
| **16** | 0.44 | 0.77 | 1.41 | 3.08 | 6.73 | 12.34 | 23.63 | 47.26 | 90.51 |
| **17** | 0.42 | 0.77 | 1.41 | 2.95 | 6.17 | 11.81 | 23.63 | 45.25 | 86.67 |
| **18** | 0.40 | 0.74 | 1.48 | 3.08 | 6.73 | 12.34 | 24.68 | 49.35 | 94.52 |
| **19** | 0.40 | 0.77 | 1.48 | 2.95 | 6.44 | 11.81 | 23.63 | 47.26 | 94.52 |
| **20** | 0.39 | 0.74 | 1.48 | 3.08 | 6.17 | 12.34 | 23.63 | 43.34 | 83.00 |
| **21** | 0.44 | 0.74 | 1.30 | 2.83 | 5.91 | 11.81 | 23.63 | 45.25 | 83.00 |
| **22** | 0.42 | 0.74 | 1.41 | 3.08 | 6.44 | 11.81 | 23.63 | 45.25 | 90.51 |
| **23** | 0.39 | 0.77 | 1.48 | 3.22 | 6.17 | 12.88 | 24.68 | 47.26 | 94.52 |
| **24** | 0.44 | 0.81 | 1.48 | 3.08 | 6.44 | 12.34 | 23.63 | 47.26 | 90.51 |
| **25** | 0.42 | 0.77 | 1.48 | 3.08 | 6.73 | 11.31 | 23.63 | 47.26 | 90.51 |
| **26** | 0.44 | 0.77 | 1.41 | 2.95 | 6.44 | 11.81 | 21.67 | 47.26 | 90.51 |
| **27** | 0.42 | 0.77 | 1.41 | 3.08 | 6.44 | 11.81 | 23.63 | 47.26 | 86.67 |
| **28** | 0.42 | 0.74 | 1.48 | 3.08 | 6.73 | 12.34 | 24.68 | 47.26 | 90.51 |
| **29** | 0.44 | 0.81 | 1.48 | 2.95 | 6.44 | 11.81 | 23.63 | 47.26 | 90.51 |
| **30** | 0.44 | 0.84 | 1.54 | 3.08 | 7.03 | 12.88 | 25.77 | 49.35 | 98.70 |
| **31** | 0.42 | 0.77 | 1.41 | 2.95 | 6.17 | 11.81 | 23.63 | 47.26 | 86.67 |
| **32** | 0.44 | 0.84 | 1.48 | 2.95 | 6.44 | 11.81 | 23.63 | 49.35 | 94.52 |
| **33** | 0.40 | 0.74 | 1.41 | 2.95 | 6.73 | 11.81 | 22.63 | 47.26 | 86.67 |
| *Note.* The frequencies are determined as the maximum likelihood estimators separating two consecutive IMFs. | | | | | | | | | |

## References

1. Juan, C. H. *et al.* Revealing the Dynamic Nature of Amplitude Modulated Neural Entrainment With Holo-Hilbert Spectral Analysis. *Front. Neurosci* **15**, 1–18 (2021).

2. Huang, N. E. *et al.* On Holo-Hilbert spectral analysis: a full informational spectral representation for nonlinear and non-stationary data. *Phil. Trans. R. Soc. A.* **374**, 20150206 (2016).

3. Canolty, R. T. &Knight, R. T. The functional role of cross-frequency coupling. *Trends. Cogn. Sci.* **14**, 506–515 (2010).

4. Flandrin, P., Rilling, G. &Gonçalvés, P. Empirical mode decomposition as a filter bank. *IEEE Signal Process. Lett.* **11**, 112–114 (2004).
